# Kidney health in the COVID-19 pandemic: An umbrella review of meta-analyses and systematic reviews

**DOI:** 10.3389/fpubh.2022.963667

**Published:** 2022-09-12

**Authors:** Letian Yang, Jian Li, Wei Wei, Cheng Yi, Yajun Pu, Ling Zhang, Tianlei Cui, Liang Ma, Juqian Zhang, Jay Koyner, Yuliang Zhao, Ping Fu

**Affiliations:** ^1^Division of Nephrology, Kidney Research Institute, West China Hospital of Sichuan University, Chengdu, China; ^2^West China School of Medicine, Sichuan University, Chengdu, China; ^3^Liverpool Centre for Cardiovascular Science, Liverpool Heart and Chest Hospital, University of Liverpool, Liverpool, United Kingdom; ^4^Division of Nephrology, Department of Medicine, University of Chicago, Chicago, IL, United States

**Keywords:** COVID-19, acute kidney injury, kidney transplant, renal replacement therapy, chronic kidney disease

## Abstract

**Background:**

This umbrella review aims to consolidate evidence from systematic reviews and meta-analyses investigating the impact of the coronavirus disease−2019 (COVID-19) on kidney health, and the associations between kidney diseases and clinical outcomes in COVID-19 patients.

**Methods:**

Five databases, namely, EMBASE, PubMed, Web of Science, the Cochrane Database of Systematic Reviews and Ovid Medline, were searched for meta-analyses and systematic reviews from January 1, 2020 to June 2, 2022. Two reviewers independently selected reviews, identified reviews for inclusion and extracted data. Disagreements were resolved by group discussions. Two reviewers independently assessed the methodological quality of all included reviews using ROBIS tool. A narrative synthesis was conducted. The characteristics and major findings of the included reviews are presented using tables and forest plots. The included meta-analyses were updated when necessary. The review protocol was prospectively registered in PROSPERO (CRD42021266300).

**Results:**

A total of 103 reviews were identified. Using ROBIS, 30 reviews were rated as low risk of bias. Data from these 30 reviews were included in the narrative synthesis. Ten meta-analyses were updated by incorporating 119 newly available cohort studies. Hospitalized COVID-19 patients had a notable acute kidney injury (AKI) incidence of 27.17%. AKI was significantly associated with mortality (pooled OR: 5.24) and severe conditions in COVID-19 patients (OR: 14.94). The pooled prevalence of CKD in COVID-19 patients was 5.7%. Pre-existing CKD was associated with a higher risk of death (pooled OR: 2.21) and disease severity (pooled OR: 1.87). Kidney transplant recipients were susceptible to SARS-CoV-2 infection (incidence: 23 per 10,000 person-weeks) with a pooled mortality of 18%.

**Conclusion:**

Kidney disease such as CKD or recipients of kidney transplants were at increased risk of contracting COVID-19. Persons with COVID-19 also had a notable AKI incidence. AKI, the need for RRT, pre-existing CKD and a history of kidney transplantation are associated with adverse outcomes in COVID-19.

**Systematic review registration:**

www.crd.york.ac.uk/prospero/display_record.php?ID=CRD42021266300, identifier: CRD42021266300.

## Introduction

The coronavirus disease−2019 (COVID-19) pandemic has caused huge challenges in healthcare globally. According to the World Health Organization (WHO), as of June 6, 2022, more than 529 million patients had been diagnosed worldwide with over 6 million COVID-19 related deaths (1). The broad clinical spectrum of COVID-19 ranges from an asymptomatic response to mild upper respiratory tract infection to critical illness with acute respiratory distress syndrome (2, 3).

Although respiratory symptoms are the dominant feature, accumulative evidence suggests that acute kidney injury (AKI) is prevalent among patients with COVID-19, particularly among critically ill patients (4–6). The presence of AKI in COVID-19 patients, particularly those with severe disease, is associated with a poor prognosis (7). A large prospective cohort study of 20,133 hospitalized COVID-19 patients noted that the mortality risk was 1.28-fold higher among CKD patients as compared to non-CKD patients (8). Recently, a rapidly growing evidence base has suggested that the presence of AKI, CKD, and other kidney impairments were associated with the poor prognosis of COVID-19 patients (1, 9–13).

Despite our understanding of COVID-19 and kidney diseases, the considerable number of studies has inevitably resulted in substantial heterogeneity in study designs and variability of risk estimates and occasionally conflicting data. When focusing on large number of meta-analyses and systematic reviews published, the highest level of evidence, the variety of evidence quality and the duplication of patient data are problematic and might hinder the identification and application of evidence-based strategies in medical practice.

Given the paucity of current knowledge, the purpose of this umbrella review of meta-analyses and systematic reviews was to summarize and consolidate evidence addressing the following two research questions: (1) what is the incidence/prevalence of AKI, CKD, and kidney transplant in COVID-19 patients? (2) what is the impact of these kidney diseases on the clinical outcomes in patients with COVID-19?

## Methods

This umbrella review was conducted following the Preferred Reporting Items for Systematic Reviews and Meta-Analyses (PRISMA) guidelines (14). The review protocol was prospectively registered in the Prospective Register of Systematic Reviews (PROSPERO, CRD42021266300).

### Objectives

To consolidate evidence to determine (1) the incidence/prevalence of AKI, CKD, and kidney transplant in COVID-19 patients and (2) the association between these kidney disorders and outcomes in patients with COVID-19.

### Study design

Meta-analyses and/or systematic reviews assessing the associations between AKI, CKD, kidney transplant, and COVID-19 were included. The diagnosis was based on 2012 Kidney Disease: Improving Global Outcomes (KDIGO) AKI and 2012 KDIGO CKD definitions. Notably, we focused on patients with a history of CKD before being diagnosed with COVID-19, instead of patients developing CKD after COVID-19- induced AKI.

The study set no restrictions on the age, sex, and ethnicity of the participants investigated, and no restriction was applied to the original recruitment locations or settings. We limited the included reviews to those published in the English language. If multiple meta-analyses and/or systematic reviews on the same research question were identified, the most recent reviews with the largest number of studies and effect sizes were included. We also assessed the quality of the included studies, synthesized the results of the included studies, and provided sufficient details of the characteristics of the included studies.

### Search strategies

Five databases, namely, EMBASE, PubMed, Web of Science, the Cochrane Database of Systematic Reviews and Ovid Medline, were systematically searched from January 1, 2020 to June 2, 2022, to identify systematic reviews and/or meta-analyses of observational studies examining the associations of kidney health with COVID-19. Appropriate free-text terms and medical subject headings (MeSH) were used to research kidney risk factors, kidney diseases, and COVID-19. The search strategy used the following terms/keywords: (“2019-nCoV” OR “Coronavirus” OR “COVID-19” OR “SARS-CoV-2” OR “2019-nCoV” OR novel coronavirus) AND (renal or kidney or nephron^*^) AND (meta-analysis or systematic review).

### Eligibility criteria

The different results from the databases were exported into EndNote X9, and duplicates were removed. Two reviewers (ZYL and LJ) independently completed the title and abstract screening in duplicate. The full texts of potentially eligible articles were scrutinized independently by the same two investigators (ZYL and LJ) to identify reviews for inclusion. Studies were included in this review if they met the inclusion criteria as follows: (1) they were systematic reviews or meta-analyses; and (2) they included observational studies which reported the associations between kidney diseases and COVID-19. Studies were excluded for the following reasons: (1) the study only reported the management or therapeutic strategy; (2) the study was an abstract only; or (3) the study was not published in English. Disagreements were resolved through discussion to reach a consensus.

### Data extraction

Two researchers (LJ and YC) independently performed the data extraction. For each eligible review, we extracted the following information: (1) name of the first author; (2) number of included studies; (3) publication year; (4) number of total participants; (5) population inclusion criteria; (6) exposures; (7) number of exposed groups; (8) controls; (9) number of controlled groups; (10) outcomes; (11) type of effect model; (12) odds ratios, risk ratios, or hazard ratios; (13) estimates; (14) 95% confidence intervals (-CIs -); (15) *I*^2^; heterogeneity (*Q*-test, *P*-value); and (16) publication bias (Egger's test, *P*-value). Disagreements were resolved by group discussion.

### Quality assessment

Two reviewers (WW and LY) critically assessed the methodological quality of all included reviews using Risk of Bias in Systematic Review (ROBIS) (15). The quality of the included reviews was assessed in the following three phases: (1) relevance of the review, (2) identifying concerns within the systematic review process under the following four domains: study eligibility criteria, identification and selection of studies, data collection and study appraisal, and synthesis and findings and (3) judging risk of bias. Each included review was given a “low,” “high” or “unclear” risk of bias score. Disagreements were resolved by group discussion.

### Data synthesis

Eligible systematic reviews and meta-analyses formed the unit of analysis. A narrative synthesis was conducted. The characteristics and major findings of the included reviews are presented using tables and forest plots.

### Update of eligible reviews

An update of an included review was necessary if meeting the following criteria: (1) The review was rated as low risk of bias using ROBIS tool; (2) there were new eligible primary studies not yet included in the existing review. If more than one reviews on the same topic were eligible, we updated the most recent review. The pooled percentages were used to meta-analyze the incidence and prevalence of outcomes. The pooled ORs with 95% CIs were used to assess the associations between exposures and clinical outcomes. A random-effects model was used to allow for heterogeneity. *P* < 0.05 was considered statistically significant. The statistical analyses were conducted in Stata, version 16.0 (Stata Corp).

## Results

### Literature search

**Figure 1:**
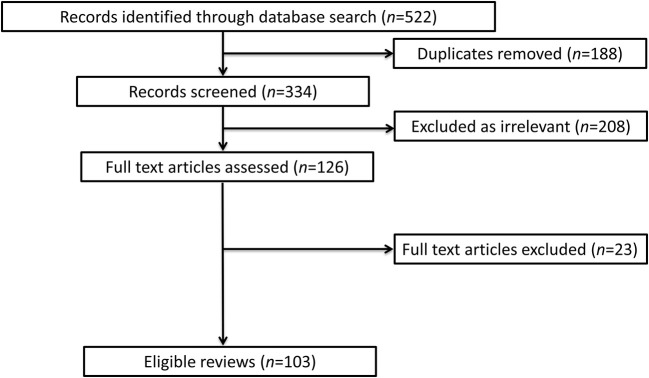
The flowchart of the study search and selection process.

Overall, the searches identified 522 studies in the five databases. After the removal of duplicates, and reviewing the titles and abstracts, 126 studies were selected for full-text screening. After applying the inclusion and exclusion criteria, 103 reviews that addressed the research questions were identified. The process of the literature search is summarized in Figure 1. The full reference list is provided in Supplementary Table 1.

### Methodological quality

Using ROBIS tool, the methodological quality of 103 included reviews was assessed. Thirty reviews (29.1%) were rated as low risk of bias, and 73 reviews (70.9%) were rated as high risk of bias. Fifty-five reviews (53.4%) did not establish the methods prior to the conduct of the review. Forty-four reviews (42.7%) did not examine if the pooled results were robust through sensitivity analysis or funnel plot. Seventy-three reviews (70.9%) did not address the bias of included primary studies. The full assessments are provided in Supplementary Table 2.

### Update of eligible reviews

One hundred and nineteen primary studies were incorporated for review update. The list was presented in Supplementary Table 3.

### Characteristics of the reviews at low risk of bias

Data from 30 reviews rated as low risk of bias were included in the narrative synthesis. The number of studies in the included reviews ranged from 6 (16) to 348 (17). Among these reviews, the earliest date of literature search was March 1, 2020 (16), and the last date of literature search was July, 2021 (18). The characteristics of these 30 reviews are shown in Table 1.

**Table 1:** The characteristics of 30 reviews at low risk of bias.

**First author**	**Publish year**	**Last date of search**	**Number of included studies**	**Sample size**	**References**
Izcovich, A.	2020	April 28, 2020	207	75,607	(19)
Mesas, A. E.	2020	July 27, 2020	60	51,225	(20)
Wang, B.	2020	March 1, 2020	6	1,558	(16)
Luo, L.	2020	July, 2020	124	NA	(21)
Lim, M. A.	2020	April 11, 2020	15	3,615	(22)
Oltean, M.	2020	June 4, 2020	12	204	(23)
Ssentongo, P.	2020	July 9, 2020	25	65,484	(24)
Hansrivijit, P.	2020	April 24, 2020	26	5,497	(25)
Zhou, S.	2020	June 16, 2020	58	13,452	(26)
Zhang, T.	2020	April 10, 2020	16	3,975	(27)
Papadopoulos, V. P.	2020	January 7, 2021	41	NA	(28)
Zhou, Y.	2020	April 26, 2020	52	21,164	(29)
Fu, E. L.	2020	May 29, 2020	142	49,048	(30)
Lee, A. C.	2021	May 25, 2020	36	22,573	(31)
Kremer, D.	2021	January 18, 2021	74	5,559	(32)
Mirjalili, H.	2021	January 10, 2020	10	11,755	(33)
Zhang, L.	2021	September 29, 2020	34	344,431	(34)
Du, P.	2021	October 22, 2020	17	7,611	(35)
Chang, R.	2021	May 1, 2020	28	12,437	(36)
Schlesinger, S.	2021	October 10, 2020	22	17,687	(37)
Menon, T.	2021	November, 2020	20	4,350	(38)
Liu, Y. F.	2021	April 13, 2020	36	6,395	(25)
Li, Y.	2021	May 2020	40	NA	(39)
Dessie, Z. G.	2021	August 31, 2020	42	423,117	(40)
Chan, K. W.	2021	October 5, 2020	74	NA	(41)
Chung, E. Y.	2021	February 22, 2021	348	1,139,979	(17)
Taylor, E. H.	2021	February 21, 2021	58	44,305	(42)
Ho, Q. Y.	2021	September 5, 2020	23	1,373	(43)
Shi, Q.	2021	July, 2021	56	79,104	(18)
Cai, X.	2021	January 30, 2021	38	42,779	(44)

### Acute kidney injury in COVID-19

#### The incidence of AKI in COVID-19 patients

Of the reviews reporting the incidence of AKI in COVID-19 patients, six were rated as low risk of bias. The findings are summarized in Table 2.

**Table 2:** The incidence of AKI in COVID-19 patients.

**Study**	**Number of included studies**	**Population**	**Incidence of AKI (95% CI)**	***I*^2^ (*p*-value)**	**References**
Zhou, S., 2020	58	All COVID-19 patients	9% (4.2–15.2%)	NA	(26)
Hansrivijit, P., 2020	26	All COVID-19 patients	8.4% (6.0–11.7%)	88.9%	(25)
		Critically ill COVID-19 patients	19.9% (11.8–31.5%)	48.4%	
		Hospitalized COVID-19 patients	7.3% (5.0–10.4%)	89.5%	
Fu, E. L., 2020	142	COVID-19 patients in Asia	5.5% (4.1–7.4%)	94%	(30)
		COVID-19 patients in the USA and Europe	28.6% (19.8–39.5%)	97%	
Chan, K. W., 2021	74	All COVID-19 patients	20.4% (12.07–28.74%)	99.72% (<0.001)	(41)
		COVID-19 patients with kidney transplant history	35.99% (26.20–45.79)	NA	
		Pediatric COVID-19 patients	16.11% (5.14–27.08)	NA	
Chung, E. Y., 2021	348	Patients with COVID-19 and CKD	7.3% (6–8.7%)	NA	(17)
Chang, R., 2021	28	COVID-19 patients admitted to ICU	32% (13–58%)	96.49 % (<0.01)	(36)

##### The overall incidence of AKI in general COVID-19 patients

Five reviews at low risk of bias reported the incidence of AKI in general COVID-19 patients. All these five reviews included hospitalized patients. The largest of which (Chan et al.) included COVID-19 patients from 17 countries (Austria, Brazil, Canada, China, Denmark, France, Germany, India, Iran, Italy, Japan, Korea, Malaysia, Spain, Turkey, United Kingdom, and United States) and suggested that the incidence of AKI in general hospitalized COVID-19 patients was 20.4% (95% CI: 12.7–28.4%) (41).

##### The incidence of AKI in severe or critically ill COVID-19 patients

Four reviews at low risk of bias reported the incidence of AKI in severe or critically-ill COVID-19 patients. The review by Chang et al. focused on 12,437 COVID-19 patients admitted to the ICU in seven countries (the USA, China, UK, Italy, Spain, France, and Mexico) and reported that the incidence of AKI was 32% (95% CI: 13–58%) (36). Hansrivijit et al. included a total of 31 studies from three countries (China, the USA, and Spain) and found that the prevalence of AKI was higher in critically ill COVID-19 patients (19.9%) than in general COVID-19 patients (7.3%) (25).

Two reviews at low risk of bias examined the association between the severity of COVID-19 and the prevalence of AKI. Liu et al. conducted the largest review (*n* = 36 studies) and reported that the incidence of AKI was significantly increased in the severe group compared with the non-severe group (OR: 11.02, 95% CI: 6.54–18.57) (45).

##### The incidence of AKI in children and adolescents with COVID-19

One review rated as low risk of bias included three studies focused on pediatric COVID-19 patients and reported that the incidence of AKI was 16.11% (41).

##### The incidence of AKI in patients with COVID-19 and pre-existing CKD

One review at low risk of bias by Chung et al. focused on new-onset AKI in patients with COVID-19 and CKD. The incidence of AKI was 73 per 1,000 person-weeks (95% CI: 60–87) (17).

##### The incidence of AKI in patients with COVID-19 from different regions

Notably, substantial difference was observed in the reported AKI incidence across regions. Chan et al. performed subgroup analyses and suggested that the incidences of AKI in Guangdong, Hong Kong, Hubei, Istanbul, Madrid, Michigan, New Delhi, New York, North Zealand, and Pennsylvania were 1.74, 3.72, 4.25, 29.17, 11.42, 44.79, 40.63, 33.07, 11.71, and 49.33%, respectively (41). In addition, Fu, E. L. included 49,048 hospitalized COVID-19 patients and reported that the incidence of AKI was 28.6% (95% CI: 19.8–39.5) in the USA and Europe (*n* = 20 studies) and 5.5% (95% CI: 4.1–7.4) in Asia (*n* = 62 studies) (30).

##### Reviews eligible for update

**Figure 2:**
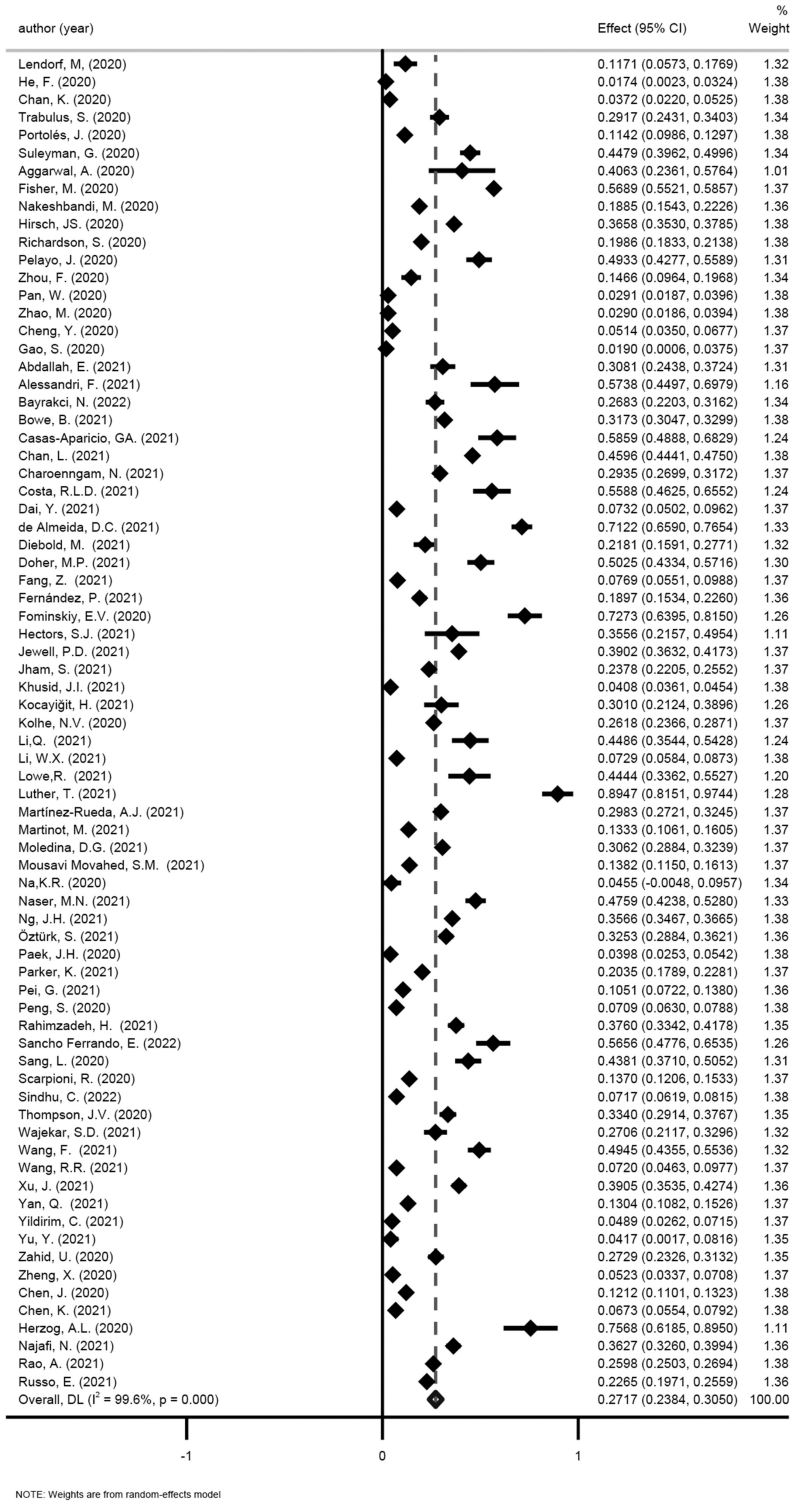
Meta-analysis of incidence of AKI in a random effect model.

One review was considered eligible for update (41), thereby, 59 newly published studies were added. Fifty-eight studies only included hospitalized patients and 1 study included both hospitalized patients and non-hospitalized patients. The pooled incidence of AKI in hospitalized COVID-19 patients was 27.17% (95% CI: 23.84–30.5%; Figure 2), while Kang et al. reported the incidence of AKI was 0.37% (95% CI: 0.25–0.55%) in all COVID-19 patients in Korean (46).

#### Risk factors for AKI in COVID-19 patients

**Figure 3:**
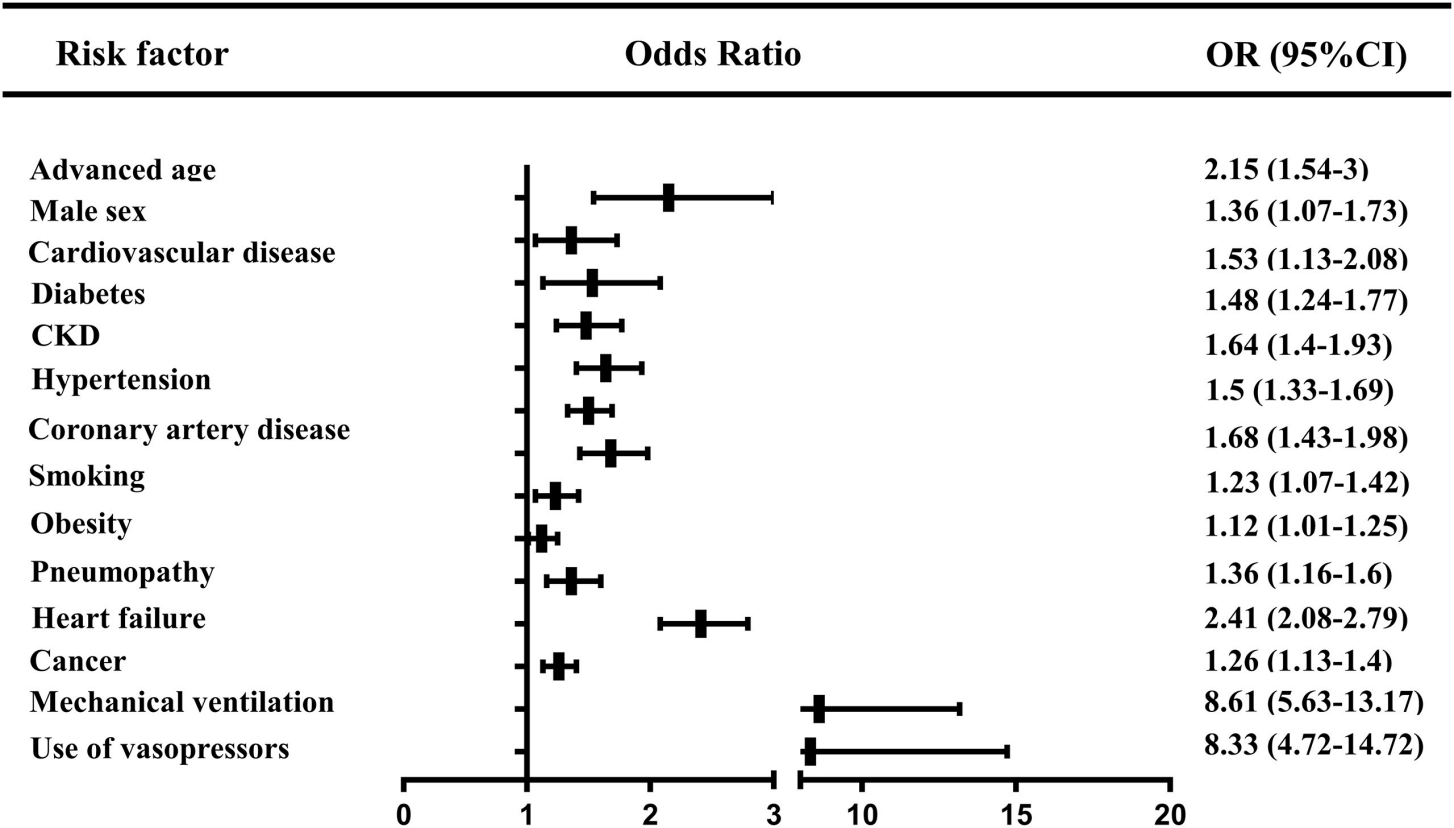
The risk factors for AKI in COVID-19 patients. CKD, chronic kidney disease.

Of the included reviews that investigated the risk factors for AKI in COVID-19 patients, three were rated as low risk of bias. These findings suggested that advanced age, male sex, smoking, obesity, comorbidities (cardiovascular disease, coronary artery disease, diabetes, CKD, hypertension, pneumopathy, heart failure, and cancer), mechanical ventilation, and the use of vasopressors were potential risk factors for AKI (Figure 3) (30, 41, 44).

#### The incidence of urgent renal replacement therapy in COVID-19

Four reviews at low risk of bias reported the incidence of urgent RRT (patients received RRT for AKI) in COVID-19 patients (Table 3), the largest by Fu et al. suggested that the incidence of RRT was 2.2% (95% CI: 1.5–3.3%) in China, and 7.7% (95% CI: 5.1–11.4%) in Europe and the USA (30). Regardless of region, Zhou et al. reported the rate of urgent-start RRT as 3.4% (95% CI: 1.9–5.4%) (26). One review rated as low risk of bias, by Brienza et al., reported that the incidence of RRT in the severe and non-severe group was 7.5 and 0.3%, respectively (OR: 14.75, 95% CI: 3.4–64.8) (47). Only one review by Chan et al. included three studies based on pediatric COVID-19 patients and suggested that the rate of RRT was 5.54% (41).

**Table 3:** The incidence of urgent RRT in COVID-19 patients.

**Study**	**Number of included studies**	**Population**	**Incidence of urgent RRT (95% CI)**	***I*^2^ (*p*-value)**	**References**
Fu, E. L., 2020	142	COVID-19 patients in China	2.2% (1.5–3.3%)	92%	(30)
		COVID-19 patients in the USA and Europe	7.7% (5.1–11.4%)	80%	
Zhou, S., 2020	58	All COVID-19 patients	3.4% (1.9–5.4%)	NA	(26)
Hansrivijit, P., 2020	26	All COVID-19 patients	3.6% (1.8–7.1%)	82.2%	(25)
Chan, K. W., 2021	74	All COVID-19 patients	2.97% (1.91–4.04%)	93.52% (<0.001)	(41)
		COVID-19 patients with kidney transplant history	12.65% (0.72–24.58)	NA	
		Pediatric COVID-19 patients	5.54% (−1.14 to 12.21)	NA	

##### Reviews eligible for update

**Figure 4:**
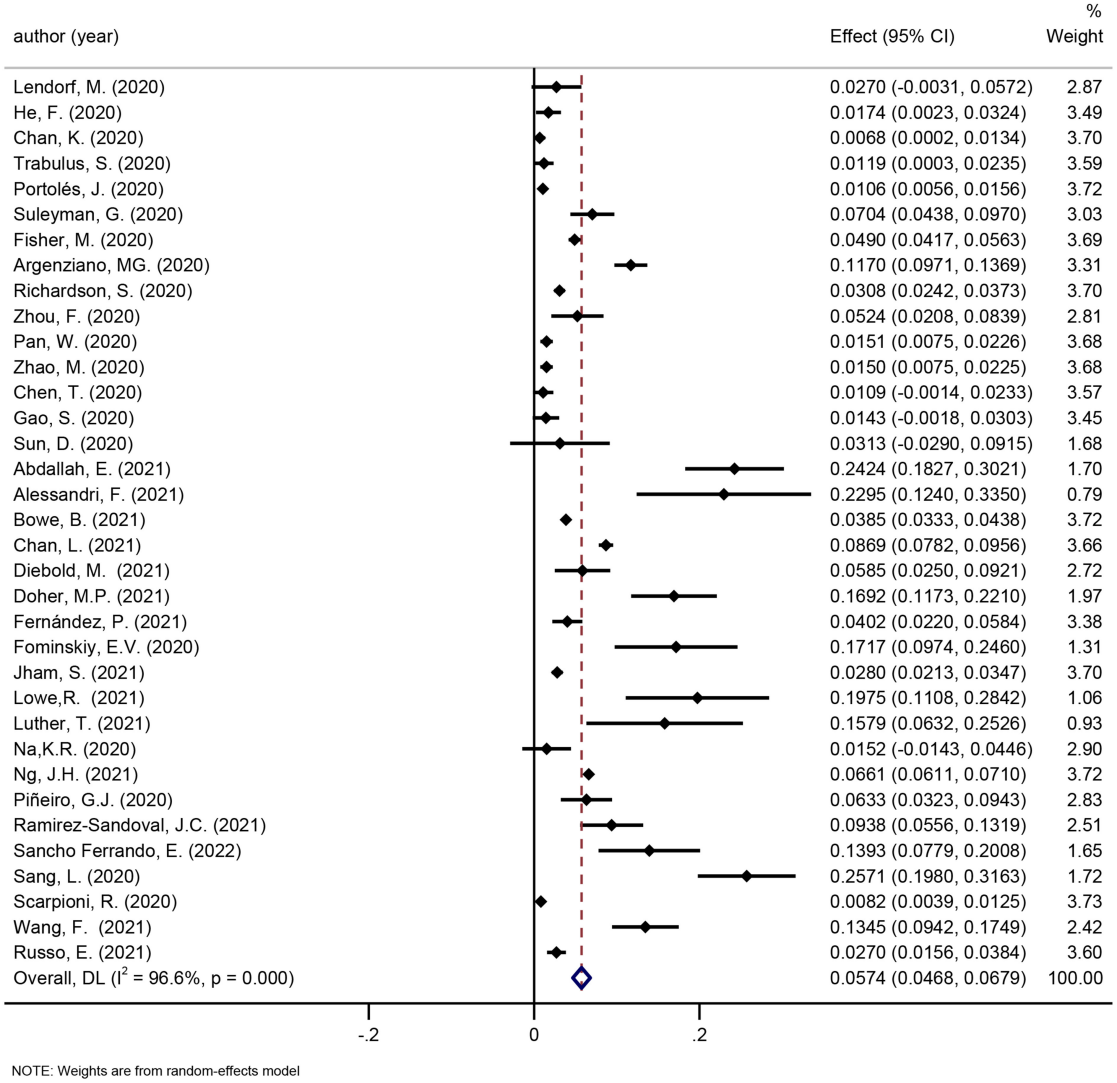
Meta-analysis of incidence of urgent RRT in a random effect model.

We considered one review eligible for update (41). Twenty newly published studies were added. Figure 4 shows the incidence of urgent RRT in COVID-19 patients (6%, 95 CI: 5–7%).

#### The predictive value of AKI and the effect of urgent RRT on poor outcomes in COVID-19 patients

Seven reviews at lower risk of bias investigated the associations between AKI and poor outcomes in COVID-19 patients, including AKI and mortality (*n* = 6), and AKI and disease severity in COVID-19 patients (*n* = 3). A summary of the findings is shown in Table 4.

**Table 4:** AKI and poor outcomes in COVID-19 patients.

**Study**	**Number of included studies**	**Exposure**	**Outcome**	**Metric**	**Effects (95% CI)**	***I*^2^ (*p*-value)**	**References**
Hansrivijit, P., 2020	26	AKI	Mortality	OR	13.33 (4.05–43.91)	85%	(25)
Lim, M. A., 2020	15	AKI	Mortality	RR	13.38 (8.15–21.95)	24% (0.25)	(22)
		AKI	Severity	RR	8.12 (4.43–14.86)	0% (0.73)	
		AKI	ICU admission	RR	5.9 (1.32–26.35)	0% (0.49)	
Zhou, Y., 2020	52	AKI	Mortality	OR	45.79 (36.88–56.85)	17% (0.31)	(29)
		AKI	Severity	OR	6.97 (3.53–13.75)	0% (0.501)	
Papadopoulos, V. P., 2020	41	AKI	Mortality	OR	7.52 (1.96–28.9)	NA	(28)
Dessie, Z. G., 2021	42	AKI	Mortality	OR	1.87 (1.48–2.26)	86.53% (<0.001)	(40)
Chang, R., 2021	28	AKI	Mortality	OR	12.47 (1.52–102.7)	81.15% (0.005)	(36)
Chan, K. W., 2021	74	AKI	Mortality	OR	9.03 (5.45–14.94)	89.7% (<0.001)	(41)
		AKI	Severity	OR	17.58 (10.51–29.38)	63% (0.004)	

##### AKI and mortality in COVID-19 patients

Of the reviews that examined the associations between AKI and mortality in COVID-19 patients, six reviews were rated as low risk of bias. The largest review by Chan et al. (*n* = 74 studies) reported that the presence of AKI was associated with an 8-fold increased risk of death in COVID-19 patients (OR: 8.33, 95% CI: 5.45–14.94) and AKI stages 1, 2, and 3 were associated with 6.5-, 23.6-, and 93.8-fold increased risks of death (41). Zhou et al. also reported that the mortality rate among people with AKI and COVID-19 was 72.3% (95% CI: 47.1–92.0%) (26). Of note, in patients with COVID-19 admitted to the ICU, AKI was significantly associated with a much elevated risk of death (OR: 12.47, 95% CI: 1.52–102.7) (36). Notably, the above results were not adjusted for confounders (such as age, sex, and comorbidities).

##### AKI and severity of COVID-19

Three reviews at low risk of bias examined the predictive value of AKI for severe conditions in COVID-19 patients. The review by Chan et al. had the largest number of included studies (*n* = 74), concluding that AKI was associated with a higher rate of ICU occupancy (OR: 17.58, 95% CI: 10.51–29.38) (41). In addition, Lim et al. reported that AKI was relevant to severe conditions (diagnosed according to the severity categories proposed by WHO) in COVID-19 patients (OR: 8.12, 95% CI: 4.43–14.86) (22). Similarly, in children and adolescents with COVID-19, Shi, Q. reported that AKI increased the risk of ICU occupancy (OR: 55.02, 95% CI: 6.26–483.35) (18). However, it is worth highlighting that above ORs were from univariate analysis without the adjustment for confounders (such as age, sex, and comorbidities).

##### Urgent RRT dependent AKI and poor outcomes of COVID-19 patients

Two reviews at low risk of bias examined the associations between RRT and poor outcomes (defined as mortality and severity or critical conditions) in COVID-19 patients. The review by Chan, K. W. with the largest number of included studies showed that the application of RRT was associated with an 18.7-fold increased risk of death and a 34-fold increased risk of critical conditions (diagnosed according to the severity categories proposed by WHO) (41). Similarly, the above results were not adjusted for any confounders.

##### Reviews eligible for update

**Figure 5:**
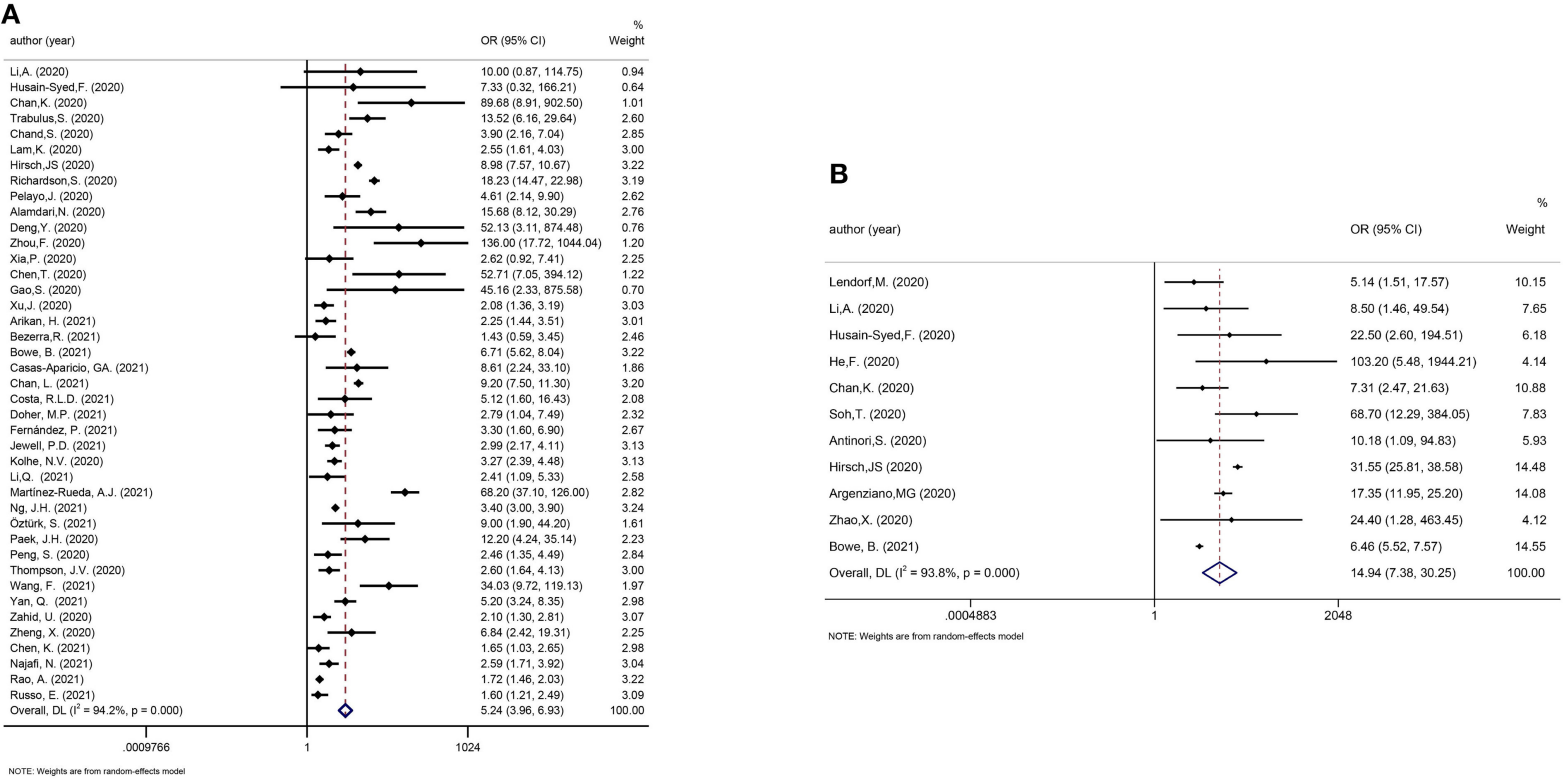
**(A)** Meta-analysis of AKI and mortality in a random effect model. **(B)** Meta-analysis of AKI and disease severity in a random effect model.

**Figure 6:**
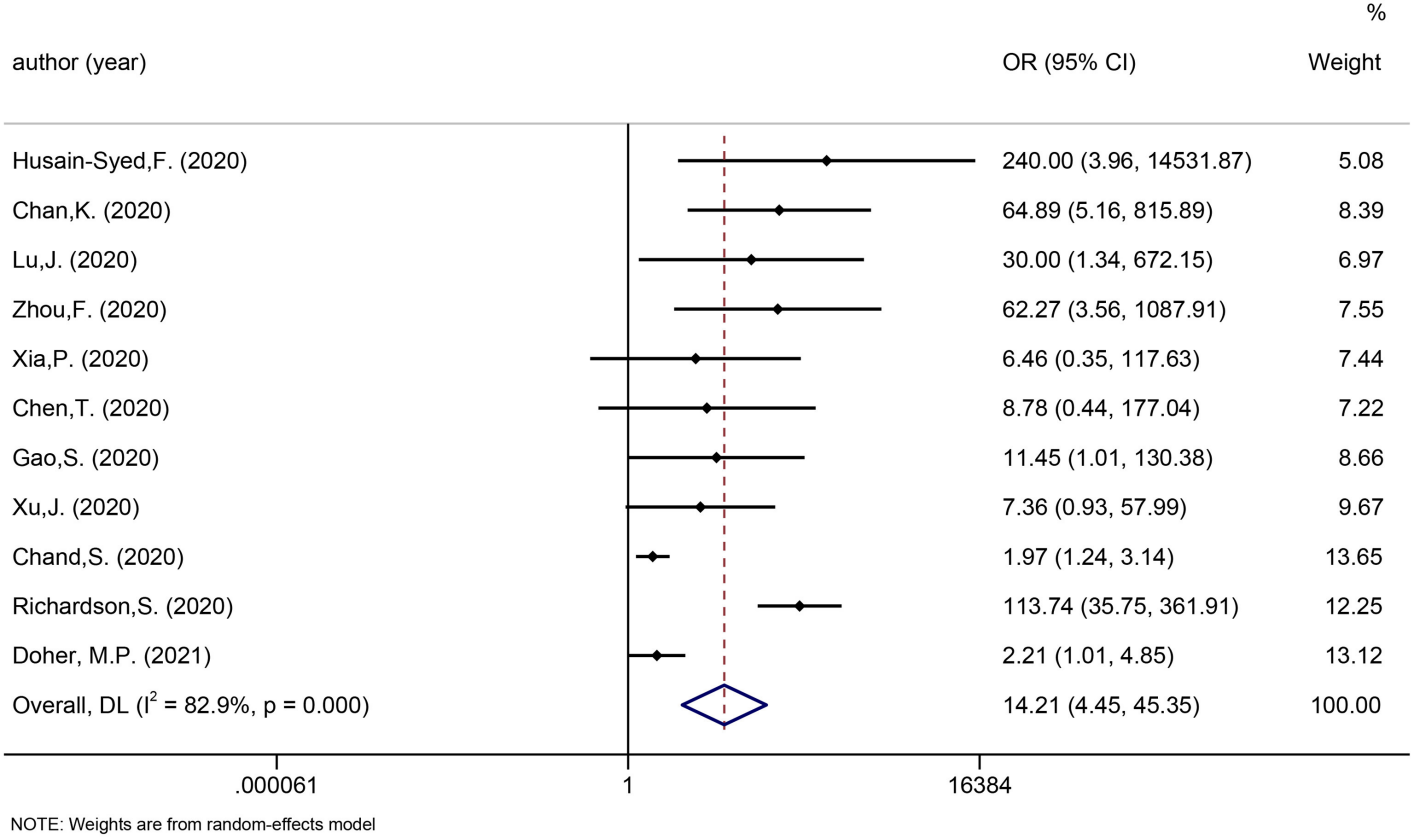
Meta-analysis of urgent RRT and mortality in a random effect model.

We considered one review eligible for update (41). Incorporating the results in the meta-analysis did not alter the significance of the associations between AKI and poor outcomes in COVID-19 patients. The updated meta-analysis showed that AKI was significantly associated with mortality and disease severity in COVID-19 patients (OR: 5.24 and 14.94, respectively; Figures 5A,B). Figure 6 shows that urgent RRT significantly predicted death in COVID-19 patients (OR: 14.21, 95% CI: 4.45–45.35).

### Chronic kidney disease in the COVID-19 pandemic

#### The incidence of COVID-19 in CKD patients

Among reviews reporting the incidence of COVID-19 in CKD patients, two reviews were rated as low risk of bias. Notably, the CKD category included in many reviews was unclear and might not be uniform across studies. The largest review by Chung et al. (*n* = 348 studies) reported that the incidence of COVID-19 in CKD patients was 66 per 1,000 person-weeks (95% CI: 58–75), and the incidence of COVID-19 varied between predialysis CKD patients and chronic dialysis patients (16 and 105 per 1,000 person-weeks, respectively) (17). Mirjalili et al. included only Iranian cases and reported that the proportion of SARS-CoV-2 infection in CKD patients was 5.0% (95% CI: 1.9–12.4%) (33).

#### Pooled prevalence of CKD in COVID-19 patients

Five reviews at low risk of bias suggested that CKD was a common comorbidity in COVID-19 patients. Zhou et al. included the largest number of studies (*n* = 37 studies), and suggested that the pooled CKD prevalence in all COVID-19 patients was 3.52% (95% CI, 1.98–5.48%) (29). Three reviews at low risk of bias summarized the prevalence of CKD in severe COVID-19 patients. Lee et al. reported that CKD was a common comorbidity in severe COVID-19 patients (8.46%, 95% CI: 3.72–18.1%) (31). Chang et al. reported that the prevalence of CKD in COVID-19 patients admitted to ICU was 9% (95%CI: 4–18%) (36). Zhou et al. suggested that CKD prevalence was higher in severe COVID-19 patients than in non-severe COVID-19 patients (OR: 3.42, 95% CI 2.05–5.61) (29). Two reviews at low risk of bias summarized the prevalence of CKD in deceased COVID-19 patients. Lee et al. reported that the proportion of CKD patients among non-survivors with COVID-19 was 9.05% (95% CI: 5.57–15.0%) (31). Zhou et al. showed that CKD was more common in deceased patients than in survivors (OR: 6.46, 95% CI: 3.40–12.29) (29). The pooled prevalence of CKD in COVID-19 patients is shown in Table 5.

**Table 5:** Pooled prevalence of CKD in COVID-19 patients.

**Study**	**Number of included studies**	**Population**	**Pooled prevalence of CKD (95% CI)**	***I*^2^ (*p*-value)**	**References**
Zhou, Y., 2020	52	All COVID-19 patients	3.52% (1.98–5.48%)	93% (<0.01)	(29)
		Severe COVID-19 patients	6.13% (2.81–10.64%)	84% (<0.01)	
		Deceased COVID-19 patients	6.36% (2.34–12.17%)	81% (<0.01)	
Lee, A. C., 2021	36	Deceased COVID-19 patients	9.028% (4.641–16.83%)	90% (<0.01)	(31)
		Severe COVID-19 patients	8.317% (3.479–18.585%)	95% (<0.01)	
Menon, T., 2021	20	All COVID-19 patients	4% (2–8%)	95% (<0.01)	(38)
Mirjalili, H., 2021	10	All COVID-19 patients	5% (1.9–12.4%)	NA	(33)
Chang, R., 2021	28	Severe COVID-19 patients	9% (4–18%)	96.97% (<0.01)	(36)

##### Reviews eligible for update

**Figure 7:**
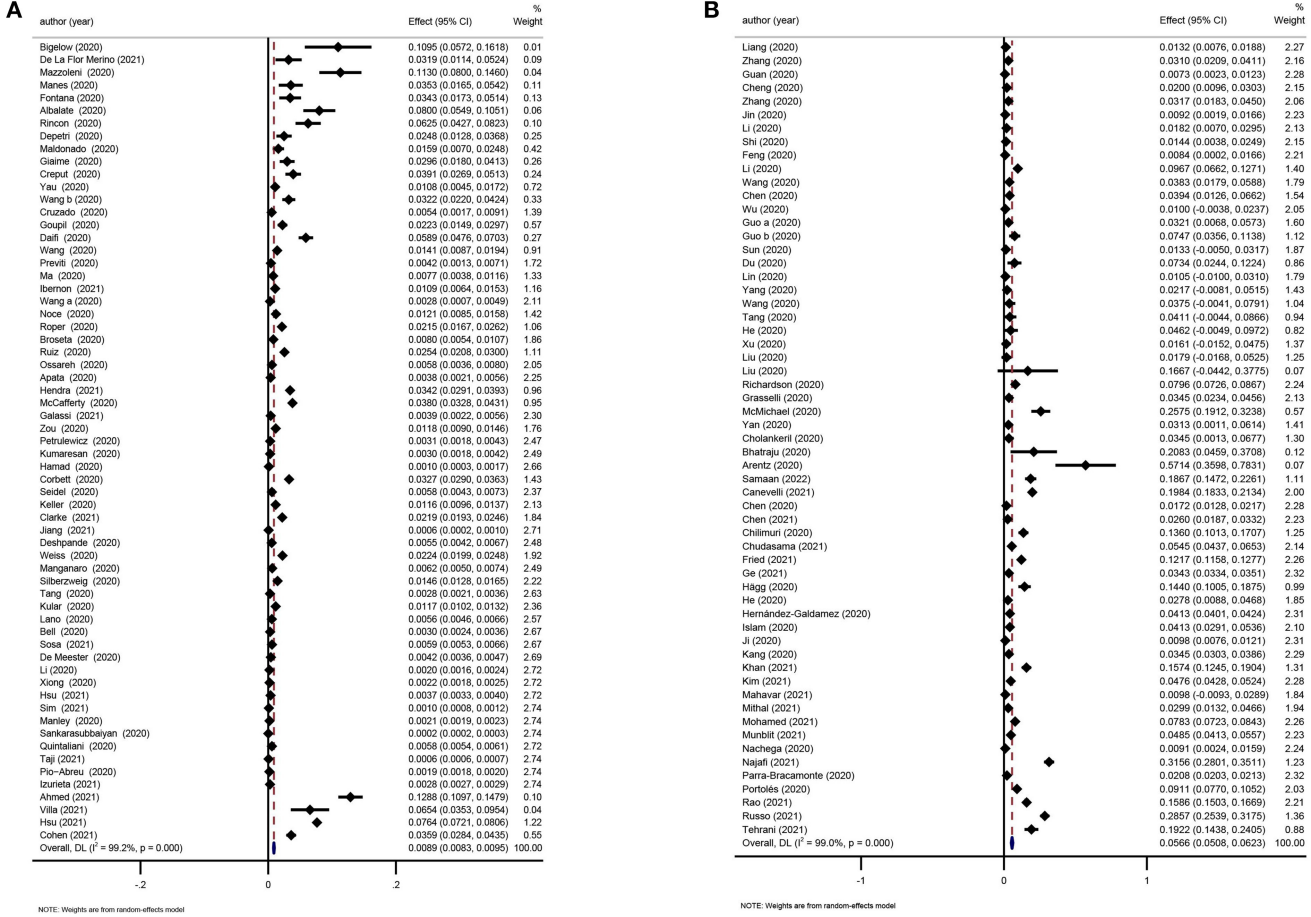
**(A)** Meta-analysis of incidence of COVID-19 in the dialysis population in a random effect model. **(B)** Meta-analysis of prevalence of CKD in COVID-19 patients in a random effect model.

Two reviews were considered eligible for updating (17, 29). The incidence of COVID-19 in the dialysis population was 0.89% (95%CI: 0.83–0.95%; Figure 7A) and the pooled prevalence of CKD in COVID-19 patients was 5.66% (95%CI: 5.08–6.23%; Figure 7B).

#### CKD and poor outcomes in COVID-19 patients

Of the included reviews focusing on CKD and mortality in COVID-19 patients, 12 reviews were rated as low risk of bias. Chung performed a review that included the largest number of studies (*n* = 348) and reported that overall mortality in patients with CKD and COVID-19 was 32 per 1,000 person-weeks (95% CI: 30–35). In different subgroups, the mortality in people with CKD5D and COVID-19 was 30 per 1,000 person-weeks (95% CI: 26–35) (17). Izcovich et al. reported a significant association between pre-existing CKD and mortality in COVID-19 patients (OR: 2.27, 95% CI: 1.69–3.05) (19). A review by Zhou, S. reported that end-stage renal disease (ESRD) was significantly associated with an increased risk of mortality in patients with COVID-19 (OR 1.81, 95% CI 1.44–2.27, *p* < 0.00001) (26). In the diabetic population with COVID-19, Schlesinger et al. found that pre-existing CKD could significantly predict the death (OR: 1.93, 95% CI: 1.28–2.90) (37).

Ten reviews rated as low risk of bias explored the associations between CKD and the severity of COVID-19. In the largest review by Izcovich et al. (*n* = 207 studies), the pooled effect showed that CKD was significantly related to an increased risk of severe COVID-19 (diagnosed according to the severity categories proposed by the WHO; OR: 2.21, 95% CI: 1.51–3.24) (19). The major findings are summarized in Table 6.

**Table 6:** CKD and poor outcomes in COVID-19.

**Study**	**Number of included studies**	**Exposure**	**Outcome**	**Metric**	**Effects (95% CI)**	***I*^2^ (*p*-value)**	**References**
Zhou, Y., 2020	52	CKD	Mortality	OR	6.46 (3.40–12.29)	1% (0.4)	(29)
		CKD	Severity	OR	3.42 (2.08–5.61)	0% (0.43)	
Izcovich, A., 2020	207	CKD	Mortality	OR	2.27 (1.69–3.05)	NA	(19)
		CKD	Severity	OR	2.21 (1.51–3.24)	NA	
Luo, L., 2020	124	CKD	Mortality	OR	3.07 (2.43–3.88)	72.9% (<0.001)	(21)
		CKD	Severity	OR	2.2 (1.27–3.80)	77.4% (<0.001)	
Mesas, A. E., 2020	60	CKD	Mortality	OR	3.2 (2.52–4.06)	75.8% (<0.001)	(20)
Ssentongo, P., 2020	25	CKD	Mortality	RR	3.25 (1.13–9.28)	84% (<0.01)	(24)
Zhou, S., 2020	58	CKD	Mortality	OR	1.97 (1.56–2.49)	65% (<0.00001)	(26)
		ESRD	Mortality	OR	1.81 (1.44–2.27)	0% (0.62)	
Zhang, T., 2020	16	CKD	Severity	OR	1.26 (0.7–2.28)	31% (0.18)	(27)
Wang, B., 2020	6	CKD	Severity	OR	2.51 (0.93–6.78)	0% (0.501)	(16)
		CKD	ICU admission	OR	2.94 (0.4–21.69)	NA	
Li, Y., 2021	40	CKD	Mortality	OR	1.57 (1.27–1.93)	62.2% (0.01)	(39)
Lee, A. C., 2021	36	CKD	Mortality	OR	8.86 (5.27–14.89)	NA	(29)
		CKD	Severity	OR	1.92 (1.65–2.23)	NA	
Zhang, L., 2021	34	CKD	Mortality	OR	8.91 (3.83–20.73)	61% (0.05)	(40)
		CKD	Severity	OR	3.2 (1.87–5.49)	0% (0.46)	
Mirjalili, H., 2021	10	CKD	Mortality	OR	0.552 (0.367–0.829)	0% (0.719)	(33)
Schlesinger, S., 2021	22	CKD	Mortality	RR	1.44 (0.96–2.15)	83%	(37)
		CKD	Severity	RR	1.93 (1.28–2.9)	81%	
Du, P., 2021	17	CKD	Severity	OR	3.59 (1.9–6.76)	19%	(35)
Liu, Y. F., 2021	36	CKD	Severity	OR	3.28 (2–5.37)	0% (0.72)	(25)
Menon, T., 2021	20	CKD	Mortality	OR	5.58 (3.27–9.54)	0% (0.84)	(38)

##### Reviews eligible for update

**Figure 8:**
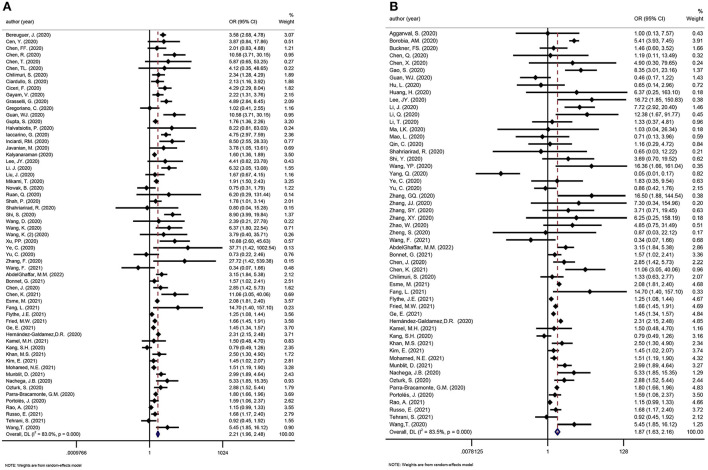
**(A)** Meta-analysis of CKD and mortality in a random effect model. **(B)** Meta-analysis of CKD and disease severity in a random effect model.

We considered one review eligible for updating (21). Evidence incorporating recent studies and the meta-analysis showed that CKD was a risk factor for death and disease deterioration in COVID-19 patients (OR: 2.21 and 1.87, respectively; Figures 8A,B).

#### The incidence of COVID-19 in kidney transplant recipients

Limited reviews have assessed the effects of COVID-19 in kidney transplant recipients (KTRs). Chung et al. included 120,281 KTRs and suggested that the incidence of COVID-19 in KTRs was 23 per 10,000 person-weeks (95% CI: 18–30) (17).

#### Adverse events in kidney transplant recipients with COVID-19

Four reviews at low risk of bias reported adverse events in KTRs with COVID-19, including AKI, urgent-RRT and mortality (Supplementary Table 4). Three reviews at low risk of bias reported the incidence of AKI in KTRs with COVID-19. The largest review by Kremer et al. included 5,559 KTRs (*n* = 74 studies) with COVID-19 and the pooled incidence of AKI was 50% (95% CI: 44–56%) (32). Two reviews at low risk of bias analyzed the application of RRT in KTRs with COVID-19. The largest review by Ho et al. (*n* = 74 studies, 1,373 KTRs with COVID-19) demonstrated that the rate of RRT was 12.4% (8.3–18%) (43). Kremer et al. reported that the mortality rate in KTRs with COVID-19 was 23% (95% CI: 20–27%). Ho et al. included 412 KTRs with COVID-19, and suggested that the proportion of critical cases was 27.7% (95% CI: 21.5–34.8%) (43).

##### Reviews eligible for update

**Figure 9:**
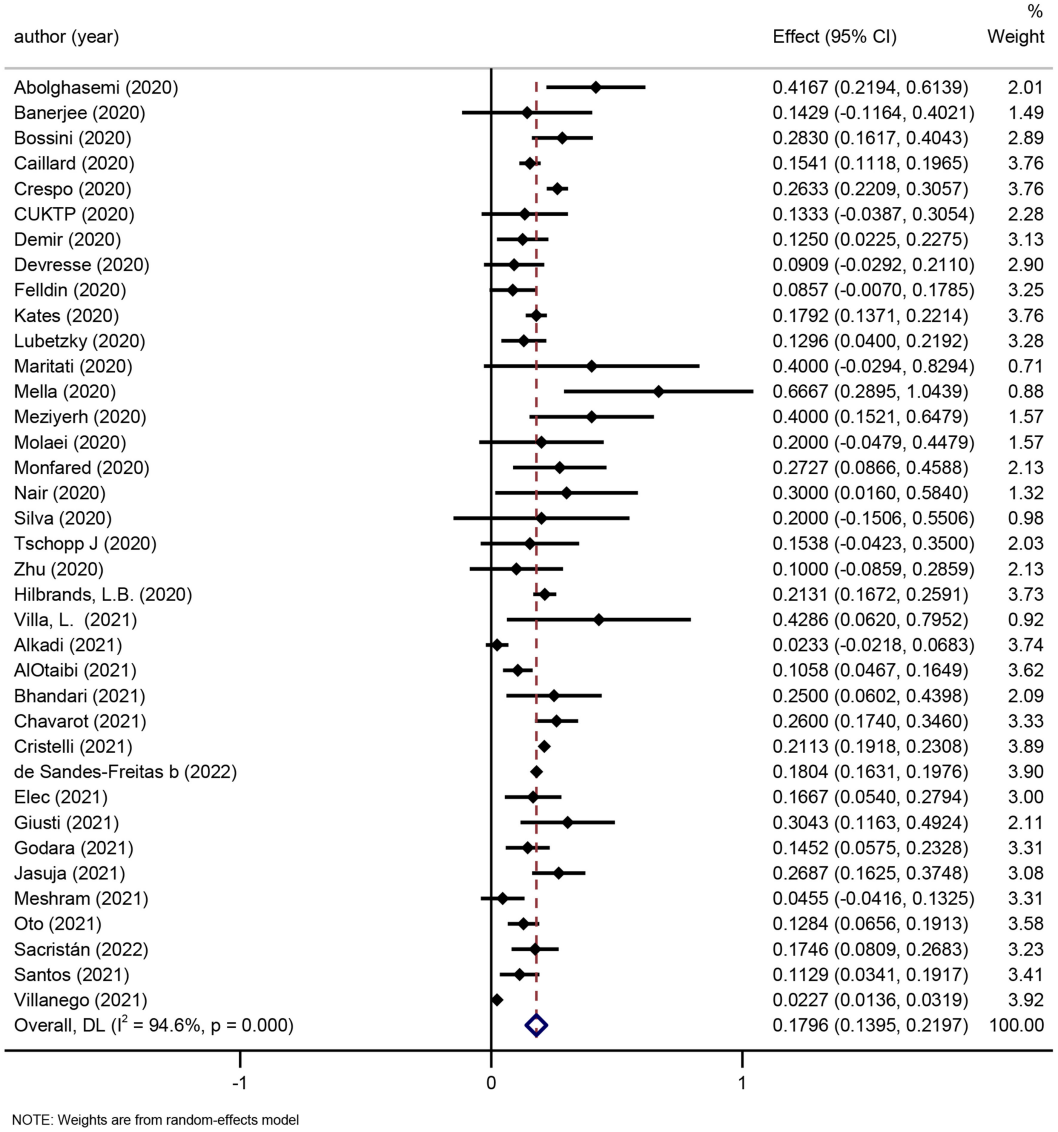
Meta-analysis of mortality in KTRs with COVID-19 in a random effect model.

We considered one review eligible for updating (32). Twenty-three recent studies examined mortality in KTRs with COVID-19. The pooled mortality rate was 18% (95% CI: 14–22%; Figure 9).

## Discussion

This umbrella review provides a comprehensive overview of existing evidence of the association between kidney diseases and COVID-19. A total of 103 systematic reviews and meta-analyses were identified, among which 30 reviews were rated as low risk of bias. We found that COVID-19 patients had a notable higher AKI incidence, varying by geographic location and disease severity. Advanced age, male sex, smoking, obesity, comorbidities (cardiovascular disease, diabetes, CKD, hypertension, pneumopathy, and cancer), mechanical ventilation, and the use of vasopressors were potential risk factors for COVID-19-associated AKI. It is important to note that many of these factors place patients at risk for other forms of AKI. The incidence of AKI, the need for RRT and pre-existing CKD were independently associated with adverse outcomes such as death and a severe disease among COVID-19 patients. KTRs are susceptible to SARS-CoV-2 infection and are at increased risk of developing a severe form of infection.

A number of studies have shown that kidney impairment is prevalent among COVID-19 patients, particularly among critically ill patients. A meta-analysis involving COVID-19 patients from 17 countries suggested that the overall incidence of AKI was 20.4% (41). In this umbrella review, the pooled incidence of AKI in COVID-19 patients was 27%. Focusing on COVID-19 patients admitted to the ICU, the incidence of AKI was 32% (36). The incidence of AKI was significantly increased in the those with severe COVID-19 group compared to those with non-severe disease (diagnosed according to the severity categories proposed by WHO), as well as in non-survivors than in survivors (45). There was also substantial heterogeneity across regions (41). The reported AKI incidence was 28.6% in the USA/Europe compared with 5.5% in China (30). In children, meta-analyses showed the incidence of AKI among pediatric COVID-19 patients was 11.9–16.11% (41, 48). The widespread application of a standardized AKI definition has facilitated comparisons across COVID-19 studies. However, as AKI is a syndrome that encompasses a multitude of clinical scenarios and pathophysiological processes, it is not surprising that the reported AKI incidence shows some variation. While the difference could partially be explained by the heterogeneities in region or patient population, other possibilities might also contribute, such as different AKI diagnostic criteria used (biochemical or coded diagnosis), hospital setting (academic center or regional hospital) and the specific definitions of relevant terms (such as baseline creatinine) (49, 50). As the pandemic goes on, the health-care community gained better understanding on the disease and are more sophisticated in treatment which might contribute to a drop of AKI and RRT rates over time (51, 52). More importantly, different research groups recently reported Omicron demonstrated lower replication in lower airway organoids, lung cells and gut cells. Whether SARS-CoV-2 variants have altered virulence on kidneys awaits further verification (53–55).

In addition to direct pathogenic mechanisms, baseline comorbidities, organ crosstalk and COVID-19 systemic effects may contribute to AKI (56). Evidence suggests that advanced age, male sex, coronary artery disease, diabetes, CKD, hypertension, elevated levels of C-reactive protein, and decreased levels of serum albumin are potential risk factors for AKI in COVID-19 patients (30, 41), which is consistent with findings reported in general hospitalized patients (57, 58). Pre-existing CKD seems to be a particularly strong risk factor for AKI. A systematic review and meta-analysis showed the new-onset AKI incidence to be 73 per 1,000 person-weeks among COVID-19 patients with CKD (17). For severely ill COVID-19 patients, critical care interventions might also be related to an increased risk of AKI, such as mechanical ventilation and the use of vasopressors (44). Observational studies can suggest an association but not causation. Since AKI patients could have an increased likelihood of being ventilated or prescribed vasopressors, caution should be exercised when interpreting these risk factors. Some additional risk factors for AKI and AKI severity, such as apolipoprotein L1 genetic variation and use of renin-angiotensin-aldosterone system inhibitors (59, 60), have been suggested by recent studies but have not yet been assessed on a meta-analysis level.

A growing number of studies have investigated the molecular mechanisms of COVID-19-induced AKI (61–65). First, the infection of SARS-CoV-2 might cause direct tubular injury. Compared with lung tissue, the kidney expresses relatively high levels of ACE2. Therefore, SARS-CoV-2 could bind to ACE2 and subsequently causes acute tubular necrosis (62). Second, also of considerable interest is the indirect tubular injury by SARS-CoV-2. Several evidence have shown that SARS-CoV-2 could attract the macrophage to infiltrate into the kidney and cause cytokine storm (63). In addition, organ crosstalk also contributes to the development of AKI, such as lung–kidney axis and cardiovascular–kidney crosstalk (64, 65).

Apart from being a target of the virus, the kidneys also seem to have a substantial influence on the outcomes of the disease. AKI has long been recognized as associated with poor outcomes. Even in non-ICU hospitalized patients with AKI, the mortality rate could reach 10–20% (66, 67). A strong and graded relationship between AKI severity and increased mortality was observed in COVID-19 patients. A meta-analysis enrolling 74 cohorts revealed that AKI was related to an 8-fold increased risk of death in COVID-19 patients. From AKI stage 1 to stage 3, the odds ratios of mortality were 6.5, 23.6, and 93.8, respectively (41). For critically ill patients in the ICU, AKI could predict an even higher risk of death (OR: 12.47, 95% CI: 1.52–102.7) (36). Meanwhile, AKI was also shown to be associated with COVID-19 severity and ICU occupancy in both adults and children (18, 22, 41). Patients with AKI who require RRT are among the most severely ill individuals in the ICU. This umbrella review indicated that AKI was significantly associated with mortality and disease severity in COVID-19 patients (OR: 5.24 and 14.94, respectively). Furthermore, the pooled rate of urgent-start RRT was to be 6% in all COVID-19 patients in this umbrella review. The application of RRT was a strong predictor of poor outcomes in COVID-19, predicting an 18.7-fold increased risk of death and a 34-fold increased risk of a critical condition (41). In Non-COVID associated AKI, prior data demonstrate that AKI was associated with an increased cost of USD $1,795 per admission, and USD $42,077 if RRT was needed (68). Affecting a large number of patients, AKI increases the risk of adverse outcomes and resource utilization, which warrants an improved strategy for the prevention, recognition and management of AKI in COVID-19 patients.

As a major chronic health burden, CKD prevalence in the general population is estimated to be between 9 and 12% (69, 70). Evidence from this umbrella review suggests that the overall CKD prevalence in COVID-19 patients is only 5.66%, which is considerably low compared to general population. CKD was more common in severely ill COVID-19 patients, with an odds ratio of 1.87 in the severe vs. non-severe group and 2.21 in the deceased vs. survivor group. Of note, CKD is a wide-spectrum clinical syndrome defined by either functional or structural abnormalities in the kidneys for more than 3 months (71). It was not clear in many of the studies if the COVID-19 patients were accurately screened for CKD as per the definition; therefore, the reported prevalence needs to be further validated. COVID-19 disproportionately affects people with chronic diseases such as CKD. The incidence of COVID-19 in people with pre-existing CKD was 66 per 10,000 person-weeks. The incidence was higher in the chronic dialysis subgroup than in the non-dialysis CKD subgroup (105 vs. 16 per 10,000 person-weeks), which may be attributable to the greater exposure to SARS-CoV-2 at health facilities when undergoing maintenance hemodialysis. In comparison, another study involving home-based dialysis patients reported a COVID-19 incidence similar to that in the general population (72). In CKD patients, COVID-19 infection was related to an increased risk of death (incidence rate ratio 10.26) compared with CKD patients without COVID-19 (58). Disrupted immune activation of both the innate and adaptive immune systems might contribute to susceptibility to infection and disease exacerbation in CKD patients (69, 73). Both CKD and ESRD were associated with increased mortality in COVID-19 patients (19, 26). Pre-existing CKD also significantly predicts the death in the diabetic COVID-19 population (37). In addition to mortality, there is also an incremental increase in the likelihood of severe COVID-19 and hospitalization in CKD patients compared with those without CKD (19, 74).

Although it remains unclear how CKD patients are more likely to contract COVID-19 and suffer from severe conditions, several reasons could partially explain these findings. First, comorbidities that accompany CKD might contribute to the development of COVID-19. In current studies, CKD was associated with multiple comorbidities, such as cardiovascular diseases and type 2 diabetes mellitus (75). A large population-based study provided robust evidence that patients with chronic heart failure had much higher risk of hospitalization of pneumonia than general population (76). Additionally, most of the patients with type 2 diabetes mellitus have abnormal immune functions, such as decreased CD3^+^T and NK T cells, and imbalance of CD4^+^ /CD8^+^ T cells, which may aggravate SARS-CoV-2 infection (77). Second, the proportion of older patients is much higher in CKD groups, which has been proved in multiple studies. Older age is a recognized risk factor for severe COVID-19. Li et al. found age older than 50 years was a feature of severe COVID-19 pneumonia (78). A retrospective study illustrated that the median age of deceased patients was 68 years ago, significantly older than recovered groups (79). Moreover, other clinical characteristics of CKD patients, such as hemodynamic instability, anemia, and electrolyte abnormality, are also possibly involved in COVID-19 development and progression.

In a state of immunocompromise, KTRs are susceptible to infections (80). This umbrella review suggested that the incidence of COVID-19 in KTRs was higher than that in the general population (23 per 10,000 person-weeks vs. 2–6 per 10,000 person-weeks) (81, 82). Because of less kidney function reserve, the use of calcineurin inhibitors and other mechanisms, AKI commonly develops in KTRs, and the external insult from COVID-19 makes these patients even more vulnerable to AKI. The pooled incidence of AKI in KTRs with COVID-19 was dramatically high (21), and AKI was associated with increased mortality in KTRs (83). Application of RRT and graft loss were relatively common in KTRs (84, 85). The pooled mortality rate was 18% in KTRs with COVID-19. KTRs are at increased risk of developing severe forms of SARS-CoV-2 infection, reflecting an increased susceptibility to COVID-19 and perhaps delayed viral clearance.

### Strength and limitations

Umbrella reviews consolidate the highest level of evidence, but there is an intrinsic limitation that they can only focus on existing meta-analyses or systematic reviews. The present umbrella review covered topics on the incidence/prevalence, aggravating factors and prognosis of AKI, CKD, and kidney transplant patients with COVID-19, while other important issues that have not yet been assessed at the meta-analysis level, such as AKI non-recovery and risk of CKD progression during post-acute COVID-19, may have been overlooked. Second, with emerging evidence, the published meta-analyses could quickly become outdated. We therefore updated five meta-analyses by incorporating 119 newly available cohort studies to guarantee that conclusions are up-to-date. The emergence of variants such as Omicron, and worldwide vaccine application are both potential major modifiers of COVID-19 epidemiology (53), however related studies focusing on kidney outcomes are scarce. Third, based on our research questions, only observational studies were available. The results were more indicative of association rather than causality, and should thus be interpreted with caution. As the included studies only performed univariate analyses, the ORs pooled were not adjusted for confounders, which is an unavoidable limitation inherited from the source studies. Fourth, notable heterogeneity existed in our sourcing reviews. For example, the criteria of COVID-19 disease severity were different across many papers looking at the same outcome. The quality of the included reviews also varied, with 73 reviews rated as high risk of bias. As such, we exclusively focused results from reviews rated as low risk of bias. It's worth mentioning that some reviews published early in the pandemic included pre-print studies to account for the rapid emerging evidence base, but results of pre-prints are subject to change after peer-review and might be a potential source of bias. Nevertheless, as we have updated these meta-analyses and enrolled only peer-reviewed articles, the pre-prints are unlikely to bias our final results. At last, therapeutic options for kidney disease patients with COVID-19 were not analyzed, as this was beyond the scope of the present umbrella review.

## Conclusion

To conclude, our umbrella review found that patients with fundamental kidney disease such as CKD and a history of kidney transplantation, were at increased risk of the development and progression of COVID-19. Persons infected by SARS-CoV-2 also had a notably high AKI incidence, with advanced age, male sex, coronary artery disease, diabetes, CKD, and hypertension being risk factors. AKI and the need for RRT were independent predictors of adverse outcomes in COVID-19. Specific observations on different SARS-CoV-2 variants and vaccination strategies, as well as follow-up studies on mid-/long-term kidney and patient outcomes in the post-acute phase of COVID-19 are needed.

## Data availability statement

The original contributions presented in the study are included in the article/Supplementary material, further inquiries can be directed to the corresponding author.

## Author contributions

LY, JL, and WW were responsible for study design, literature research, study selection, and manuscript drafting. YZ was responsible for study design, statistical analysis, and manuscript drafting. CY and YP were responsible for data extraction. JK and JZ were responsible for manuscript revision. LZ and LM were responsible for data verification and manuscript revision. PF and TC were responsible for the study design and manuscript revision. All authors contributed to the article and approved the submitted version.

## Funding

This study was supported by Science and Technology Department of Sichuan Province (2021YJ0423) and 135 project for disciplines of excellence, West China Hospital, Sichuan University (2020HXFH014).

## Conflict of interest

The authors declare that the research was conducted in the absence of any commercial or financial relationships that could be construed as a potential conflict of interest.

## Publisher's note

All claims expressed in this article are solely those of the authors and do not necessarily represent those of their affiliated organizations, or those of the publisher, the editors and the reviewers. Any product that may be evaluated in this article, or claim that may be made by its manufacturer, is not guaranteed or endorsed by the publisher.

## References

[1] Zheng X Yang H Li X Li H Xu L Yu Q . Prevalence of kidney injury and associations with critical illness and death in patients with COVID-19. Clin J Am Soc Nephrol. (2020) 15:1549–56. 10.2215/CJN.04780420 32943396 PMC7646240

[2] Zhu N Zhang D Wang W Li X Yang B Song J . A novel coronavirus from patients with pneumonia in China, 2019. N Engl J Med. (2020) 382:727–33. 10.1056/NEJMoa2001017 31978945 PMC7092803

[3] Wang D Hu B Hu C Zhu F Liu X Zhang J . Clinical characteristics of 138 hospitalized patients with 2019 novel coronavirus-infected pneumonia in Wuhan, China. J Am Med Assoc. (2020) 323:1061–9. 10.1001/jama.2020.1585 32031570 PMC7042881

[4] Richardson S Hirsch JS Narasimhan M Crawford JM McGinn T Davidson KW . Presenting characteristics, comorbidities, and outcomes among 5700 patients hospitalized with COVID-19 in the New York City Area. J Am Med Assoc. (2020) 323:2052–9. 10.1001/jama.2020.6775 32320003 PMC7177629

[5] Argenziano MG Bruce SL Slater CL Tiao JR Baldwin MR Barr RG . Characterization and clinical course of 1000 patients with coronavirus disease 2019 in New York: retrospective case series. BMJ. (2020) 369:m1996. 10.1136/bmj.m1996 32471884 PMC7256651

[6] Hirsch JS Ng JH Ross DW Sharma P Shah HH Barnett RL . Acute kidney injury in patients hospitalized with COVID-19. Kidney Int. (2020) 98:209–18. 10.1016/j.kint.2020.05.006 32416116 PMC7229463

[7] Bruchfeld A. The COVID-19 pandemic: consequences for nephrology. Nat Rev Nephrol. (2021) 17:81–2. 10.1038/s41581-020-00381-4 33257872 PMC7703720

[8] Docherty AB Harrison EM Green CA Hardwick HE Pius R Norman L . Features of 20 133 UK patients in hospital with covid-19 using the ISARIC WHO Clinical Characterisation Protocol: prospective observational cohort study. BMJ. (2020) 369:m1985. 10.1136/bmj.m1985 32444460 PMC7243036

[9] Sullivan MK Lees JS Drake TM Docherty AB Oates G Hardwick HE . Acute kidney injury in patients hospitalized with COVID-19 from the ISARIC WHO CCP-UK study: a prospective, multicentre cohort study. Nephrol Dial Transplant. (2022) 37:271–84. 10.1093/ndt/gfab303 34661677 PMC8788218

[10] Kolhe NV Fluck RJ Selby NM Taal MW. Acute kidney injury associated with COVID-19: a retrospective cohort study. PLoS Med. (2020) 17:e1003406. 10.1371/journal.pmed.1003406 33125416 PMC7598516

[11] Ng JH Hirsch JS Hazzan A Wanchoo R Shah HH Malieckal DA . Outcomes among patients hospitalized with COVID-19 and acute kidney injury. Am J Kidney Dis. (2021) 77:204–15.e1. 10.1053/j.ajkd.2020.09.002 32961245 PMC7833189

[12] Pakhchanian H Raiker R Mukherjee A Khan A Singh S Chatterjee A. Outcomes of COVID-19 in CKD patients: a multicenter electronic medical record cohort study. Clin J Am Soc Nephrol. (2021) 16:785–6. 10.2215/CJN.13820820 33558255 PMC8259489

[13] Weinhandl ED Wetmore JB Peng Y Liu J Gilbertson DT Johansen KL. Initial effects of COVID-19 on patients with ESKD. J Am Soc Nephrol. (2021) 32:1444–53. 10.1681/ASN.2021010009 33833076 PMC8259631

[14] Liberati A Altman DG Tetzlaff J Mulrow C Gøtzsche PC Ioannidis JP . The PRISMA statement for reporting systematic reviews and meta-analyses of studies that evaluate healthcare interventions: explanation and elaboration. BMJ. (2009) 339:b2700. 10.1136/bmj.b2700 19622552 PMC2714672

[15] Whiting P Savović J Higgins JP Caldwell DM Reeves BC Shea B . ROBIS: a new tool to assess risk of bias in systematic reviews was developed. J Clin Epidemiol. (2016) 69:225–34. 10.1016/j.jclinepi.2015.06.005 26092286 PMC4687950

[16] Wang B Li R Lu Z Huang Y. Does comorbidity increase the risk of patients with COVID-19: evidence from meta-analysis. Aging. (2020) 12:6049–57. 10.18632/aging.103000 32267833 PMC7185114

[17] Chung EY Palmer SC Natale P Krishnan A Cooper TE Saglimbene VM . Incidence and outcomes of COVID-19 in people with CKD: a systematic review and meta-analysis. Am J Kidney Dis. (2021) 7:3. 10.1053/j.ajkd.2021.07.003 34364906 PMC8339603

[18] Shi Q Wang Z Liu J Wang X Zhou Q Li Q . Risk factors for poor prognosis in children and adolescents with COVID-19: a systematic review and meta-analysis. EClinicalMedicine. (2021) 41:101155. 10.1016/j.eclinm.2021.101155 34693233 PMC8523335

[19] Izcovich A Ragusa MA Tortosa F Lavena Marzio MA Agnoletti C Bengolea A . Prognostic factors for severity and mortality in patients infected with COVID-19: a systematic review. PLoS ONE. (2020) 15:e0241955. 10.1371/journal.pone.0241955 33201896 PMC7671522

[20] Mesas AE Cavero-Redondo I Álvarez-Bueno C Sarriá Cabrera MA Maffei de Andrade S Sequí-Dominguez I . Predictors of in-hospital COVID-19 mortality: a comprehensive systematic review and meta-analysis exploring differences by age, sex and health conditions. PLoS ONE. (2020) 15:e0241742. 10.1371/journal.pone.0241742 33141836 PMC7608886

[21] Luo L Fu M Li Y Hu S Luo J Chen Z . The potential association between common comorbidities and severity and mortality of coronavirus disease 2019: a pooled analysis. Clin Cardiol. (2020) 43:1478–93. 10.1002/clc.23465 33026120 PMC7675427

[22] Lim MA Pranata R Huang I Yonas E Soeroto AY Supriyadi R. Multiorgan failure with emphasis on acute kidney injury and severity of COVID-19: systematic review and meta-analysis. Can J Kidney Health Dis. (2020) 7:2054358120938573. 10.1177/2054358120938573 32685180 PMC7343353

[23] Oltean M Søfteland JM Bagge J Ekelund J Felldin M Schult A . Covid-19 in kidney transplant recipients: a systematic review of the case series available three months into the pandemic. Infect Dis. (2020) 52:830–7. 10.1080/23744235.2020.1792977 32657186

[24] Ssentongo P Ssentongo AE Heilbrunn ES Ba DM Chinchilli VM. Association of cardiovascular disease and 10 other pre-existing comorbidities with COVID-19 mortality: a systematic review and meta-analysis. PLoS ONE. (2020) 15:e0238215. 10.1371/journal.pone.0238215 32845926 PMC7449476

[25] Hansrivijit P Qian C Boonpheng B Thongprayoon C Vallabhajosyula S Cheungpasitporn W . Incidence of acute kidney injury and its association with mortality in patients with COVID-19: a meta-analysis. J Investig Med. (2020) 68:1261–70. 10.1136/jim-2020-001407 32655013

[26] Zhou S Xu J Xue C Yang B Mao Z Ong ACM. Coronavirus-associated kidney outcomes in COVID-19, SARS, and MERS: a meta-analysis and systematic review. Ren Fail. (2020) 43:1–15. 10.1080/0886022X.2020.1847724 33256491 PMC7717867

[27] Zhang T Huang WS Guan W Hong Z Gao J Gao G . Risk factors and predictors associated with the severity of COVID-19 in China: a systematic review, meta-analysis, and meta-regression. J Thorac Dis. (2020) 12:7429–41. 10.21037/jtd-20-1743 33447431 PMC7797827

[28] Papadopoulos VP Koutroulos MV Zikoudi DG Bakola SA Avramidou P Touzlatzi N . Diabetes-related acute metabolic emergencies in COVID-19 patients: a systematic review and meta-analysis. Diabetol Int. (2021) 2021:1–15. 10.1101/2021.01.12.21249697 33777611 PMC7985576

[29] Zhou Y Ren Q Chen G Jin Q Cui Q Luo H . Chronic kidney diseases and acute kidney injury in patients with COVID-19: evidence from a meta-analysis. Front Med. (2020) 7:588301. 10.3389/fmed.2020.588301 33224965 PMC7670057

[30] Fu EL Janse RJ de Jong Y van der Endt VHW Milders J van der Willik EM . Acute kidney injury and kidney replacement therapy in COVID-19: a systematic review and meta-analysis. Clin Kidney J. (2020) 13:550–63. 10.1093/ckj/sfaa160 32897278 PMC7467593

[31] Lee AC Li WT Apostol L Ma J Taub PR Chang EY . Cardiovascular, cerebrovascular, and renal co-morbidities in COVID-19 patients: a systematic-review and meta-analysis. Comput Struct Biotechnol J. (2021) 19:3755–64. 10.1016/j.csbj.2021.06.038 34221254 PMC8238636

[32] Kremer D Pieters TT Verhaar MC Berger SP Bakker SJL van Zuilen AD . A systematic review and meta-analysis of COVID-19 in kidney transplant recipients: lessons to be learned. Am J Transplant. (2021) 2021:ajt.16742. 10.1111/ajt.16742 34212499 PMC9292797

[33] Mirjalili H Dastgheib SA Shaker SH Bahrami R Mazaheri M Sadr-Bafghi SMH . Proportion and mortality of Iranian diabetes mellitus, chronic kidney disease, hypertension and cardiovascular disease patients with COVID-19: a meta-analysis. J Diabetes Metab Disord. (2021) 20:1–13. 10.1007/s40200-021-00768-5 33654683 PMC7907796

[34] Zhang L Hou J Ma FZ Li J Xue S Xu ZG. The common risk factors for progression and mortality in COVID-19 patients: a meta-analysis. Arch Virol. (2021) 166:2071–87. 10.1007/s00705-021-05012-2 33797621 PMC8017903

[35] Du P Li D Wang A Shen S Ma Z Li X . Systematic review and meta-analysis of risk factors associated with severity and death in COVID-19 patients. Can J Infect Dis Medical Microbiol. (2021) 2021:6660930. 10.1155/2021/6660930 33936349 PMC8040926

[36] Chang R Elhusseiny KM Yeh YC Sun WZ. COVID-19 ICU and mechanical ventilation patient characteristics and outcomes-a systematic review and meta-analysis. PLoS ONE. (2021) 16:e0246318. 10.1371/journal.pone.0246318 33571301 PMC7877631

[37] Schlesinger S Neuenschwander M Lang A Pafili K Kuss O Herder C . Risk phenotypes of diabetes and association with COVID-19 severity and death: a living systematic review and meta-analysis. Diabetologia. (2021) 64:1480–91. 10.1007/s00125-021-05458-8 33907860 PMC8079163

[38] Menon T Sharma R Kataria S Sardar S Adhikari R Tousif S . The association of acute kidney injury with disease severity and mortality in COVID-19: a systematic review and meta-analysis. Cureus. (2021) 13:e13894. 10.7759/cureus.13894 33880250 PMC8045562

[39] Li Y Ashcroft T Chung A Dighero I Dozier M Horne M . Risk factors for poor outcomes in hospitalised COVID-19 patients: a systematic review and meta-analysis. J Glob Health. (2021) 11:10001. 10.7189/jogh.11.10001 33767855 PMC7980087

[40] Dessie ZG Zewotir T. Mortality-related risk factors of COVID-19: a systematic review and meta-analysis of 42 studies and 423,117 patients. BMC Infect Dis. (2021) 21:855. 10.1186/s12879-021-06536-3 34418980 PMC8380115

[41] Chan KW Yu KY Lee PW Lai KN Tang SC. Global REnal Involvement of CORonavirus Disease 2019 (RECORD): a systematic review and meta-analysis of incidence, risk factors, and clinical outcomes. Front Med. (2021) 8:678200. 10.3389/fmed.2021.678200 34113640 PMC8185046

[42] Taylor EH Marson EJ Elhadi M Macleod KDM Yu YC Davids R . Factors associated with mortality in patients with COVID-19 admitted to intensive care: a systematic review and meta-analysis. Anaesthesia. (2021) 76:1224–32. 10.1111/anae.15532 34189735 PMC8444810

[43] Ho QY Sultana R Lee TL Thangaraju S Kee T Htay H. Coronavirus disease 2019 in kidney transplant recipients: a systematic review and meta-analysis. Singapore Med J. (2021) 2021:171. 10.11622/smedj.2021171 34688231 PMC10645004

[44] Cai X Wu G Zhang J Yang L. Risk factors for acute kidney injury in adult patients with COVID-19: a systematic review and meta-analysis. Front Med. (2021) 8:719472. 10.3389/fmed.2021.719472 34938742 PMC8685316

[45] Liu YF Zhang Z Pan XL Xing GL Zhang Y Liu ZS . The chronic kidney disease and acute kidney injury involvement in COVID-19 pandemic: a systematic review and meta-analysis. PLoS ONE. (2021) 16:e0244779. 10.1371/journal.pone.0244779 33400721 PMC7785235

[46] Khan MS Dogra R Miriyala LKV Salman FNU Ishtiaq R Patti DK . Clinical characteristics and outcomes of patients with Corona Virus Disease 2019 (COVID-19) at Mercy Health Hospitals, Toledo, Ohio. PLoS ONE. (2021) 16:e0250400. 10.1371/journal.pone.0250400 33886663 PMC8061926

[47] Brienza N Puntillo F Romagnoli S Tritapepe L. Acute kidney injury in coronavirus disease 2019 infected patients: a meta-analytic study. Blood Purif. (2021) 50:35–41. 10.1159/000509274 32615555 PMC7445379

[48] Aronoff SC Hall A Del Vecchio MT. The natural history of severe acute respiratory syndrome coronavirus 2-related multisystem inflammatory syndrome in children: a systematic review. J Pediatric Infect Dis Soc. (2020) 9:746–51. 10.1093/jpids/piaa112 32924059 PMC7797745

[49] Lafrance JP Miller DR. Defining acute kidney injury in database studies: the effects of varying the baseline kidney function assessment period and considering CKD status. Am J Kidney Dis. (2010) 56:651–60. 10.1053/j.ajkd.2010.05.011 20673605

[50] Hoste EAJ Kellum JA Selby NM Zarbock A Palevsky PM Bagshaw SM . Global epidemiology and outcomes of acute kidney injury. Nat Rev Nephrol. (2018) 14:607–25. 10.1038/s41581-018-0052-0 30135570

[51] Khusid JA Becerra AZ Gallante B Sadiq AS Atallah WM Badani KK . Cancer, mortality, and acute kidney injury among hospitalized patients with SARS-CoV-2 infection. Asian Pac J Cancer Prev. (2021) 22:517–22. 10.31557/APJCP.2021.22.2.517 33639668 PMC8190375

[52] Martinot M Eyriey M Gravier S Bonijoly T Kayser D Ion C . Predictors of mortality, ICU hospitalization, and extrapulmonary complications in COVID-19 patients. Infect Dis Now. (2021) 51:518–25. 10.1016/j.idnow.2021.07.002 34242842 PMC8260549

[53] Meng B Abdullahi A Ferreira I Goonawardane N Saito A Kimura I . Altered TMPRSS2 usage by SARS-CoV-2 Omicron impacts tropism and fusogenicity. Nature. (2022) 603:706–14. 10.1038/s41586-022-04474-x 35104837 PMC8942856

[54] Hui KPY Ho JCW Cheung MC Ng KC Ching RHH Lai KL . SARS-CoV-2 Omicron variant replication in human bronchus and lung *ex vivo*. Nature. (2022) 2022:6. 10.1038/s41586-022-04479-6 35104836

[55] Suzuki R Yamasoba D Kimura I Wang L Kishimoto M Ito J . Attenuated fusogenicity and pathogenicity of SARS-CoV-2 Omicron variant. Nature. (2022) 603:700–5. 10.1038/s41586-022-04462-1 35104835 PMC8942852

[56] Nadim MK Forni LG Mehta RL Connor MJ Liu KD Ostermann M . COVID-19-associated acute kidney injury: consensus report of the 25th Acute Disease Quality Initiative (ADQI) Workgroup. Nat Rev Nephrol. (2020) 16:747–64. 10.1038/s41581-020-00356-5 33060844 PMC7561246

[57] Yu MY Lee SW Baek SH Na KY Chae DW Chin HJ . Hypoalbuminemia at admission predicts the development of acute kidney injury in hospitalized patients: a retrospective cohort study. PLoS One. (2017) 12:e0180750. 10.1371/journal.pone.0180750 28723973 PMC5516984

[58] Roberts G Phillips D McCarthy R Bolusani H Mizen P Hassan M . Acute kidney injury risk assessment at the hospital front door: what is the best measure of risk? Clin Kidney J. (2015) 8:673–80. 10.1093/ckj/sfv080 26613022 PMC4655789

[59] Hung AM Shah SC Bick AG Yu Z Chen HC Hunt CM . APOL1 risk variants, acute kidney injury, and death in participants with African ancestry hospitalized with COVID-19 from the million veteran program. J Am Med Assoc Intern Med. (2022) 182:386–95. 10.1001/jamainternmed.2021.8538 35089317 PMC8980930

[60] Birkelo BC Parr SK Perkins AM Greevy RA Arroyo JP Hung AM . Renin-angiotensin-aldosterone system inhibitors and the risk of AKI in COVID-19 compared with influenza. Clin J Am Soc Nephrol. (2022) 17:423–5. 10.2215/CJN.11190821 35110376 PMC8975037

[61] Ahmadian E Hosseiniyan Khatibi SM Razi Soofiyani S Abediazar S Shoja MM Ardalan M . Covid-19 and kidney injury: pathophysiology and molecular mechanisms. Rev Med Virol. (2021) 31:e2176. 10.1002/rmv.2176 33022818 PMC7646060

[62] Soleimani M. Acute kidney injury in SARS-CoV-2 infection: direct effect of virus on kidney proximal tubule cells. Int J Mol Sci. (2020) 21:932. 10.3390/ijms21093275 32380787 PMC7247357

[63] Fu Y Cheng Y Wu Y. Understanding SARS-CoV-2-mediated inflammatory responses: from mechanisms to potential therapeutic tools. Virol Sin. (2020) 35:266–71. 10.1007/s12250-020-00207-4 32125642 PMC7090474

[64] Panitchote A Mehkri O Hastings A Hanane T Demirjian S Torbic H . Factors associated with acute kidney injury in acute respiratory distress syndrome. Ann Intensive Care. (2019) 9:74. 10.1186/s13613-019-0552-5 31264042 PMC6603088

[65] Ronco C Bellasi A Di Lullo L. Cardiorenal syndrome: an overview. Adv Chronic Kidney Dis. (2018) 25:382–90. 10.1053/j.ackd.2018.08.004 30309455

[66] Selby NM Crowley L Fluck RJ McIntyre CW Monaghan J Lawson N . Use of electronic results reporting to diagnose and monitor AKI in hospitalized patients. Clin J Am Soc Nephrol. (2012) 7:533–40. 10.2215/CJN.08970911 22362062

[67] Uchino S Bellomo R Bagshaw SM Goldsmith D. Transient azotaemia is associated with a high risk of death in hospitalized patients. Nephrol Dial Transplant. (2010) 25:1833–9. 10.1093/ndt/gfp624 20054022

[68] Silver SA Long J Zheng Y Chertow GM. Cost of acute kidney injury in hospitalized patients. J Hosp Med. (2017) 12:70–6. 10.12788/jhm.2683 28182800

[69] Jdiaa SS Mansour R El Alayli A Gautam A Thomas P Mustafa RA. COVID-19 and chronic kidney disease: an updated overview of reviews. J Nephrol. (2022) 2022:1–17. 10.1007/s40620-021-01206-8 35013985 PMC8747880

[70] GBD Chronic Kidney Disease Collaboration. Global, regional, and national burden of chronic kidney disease, 1990–2017: a systematic analysis for the Global Burden of Disease Study 2017. Lancet. (2020) 395:709–33. 10.1016/S0140-6736(20)30045-3 32061315 PMC7049905

[71] Levin A Stevens PE. Summary of KDIGO 2012 CKD guideline: behind the scenes, need for guidance, and a framework for moving forward. Kidney Int. (2014) 85:49–61. 10.1038/ki.2013.444 24284513

[72] Jiang HJ Tang H Xiong F Chen WL Tian JB Sun J . COVID-19 in peritoneal dialysis patients. Clin J Am Soc Nephrol. (2020) 16:121–3. 10.2215/CJN.07200520 32900690 PMC7792636

[73] Kato S Chmielewski M Honda H Pecoits-Filho R Matsuo S Yuzawa Y . Aspects of immune dysfunction in end-stage renal disease. Clin J Am Soc Nephrol. (2008) 3:1526–33. 10.2215/CJN.00950208 18701615 PMC4571158

[74] Oetjens MT Luo JZ Chang A Leader JB Hartzel DN Moore BS . Electronic health record analysis identifies kidney disease as the leading risk factor for hospitalization in confirmed COVID-19 patients. PLoS ONE. (2020) 15:e0242182. 10.1371/journal.pone.0242182 33180868 PMC7660530

[75] Viasus D Garcia-Vidal C Cruzado JM Adamuz J Verdaguer R Manresa F . Epidemiology, clinical features and outcomes of pneumonia in patients with chronic kidney disease. Nephrol Dial Transplant. (2011) 26:2899–906. 10.1093/ndt/gfq798 21273232

[76] Mor A Thomsen RW Ulrichsen SP Sørensen HT. Chronic heart failure and risk of hospitalization with pneumonia: a population-based study. Eur J Intern Med. (2013) 24:349–53. 10.1016/j.ejim.2013.02.013 23510659

[77] Allard R Leclerc P Tremblay C Tannenbaum TN. Diabetes and the severity of pandemic influenza A (H1N1) infection. Diabetes Care. (2010) 33:1491–3. 10.2337/dc09-2215 20587722 PMC2890346

[78] Li K Wu J Wu F Guo D Chen L Fang Z . The clinical and chest CT features associated with severe and critical COVID-19 pneumonia. Invest Radiol. (2020) 55:327–31. 10.1097/RLI.0000000000000672 32118615 PMC7147273

[79] Chen T Wu D Chen H Yan W Yang D Chen G . Clinical characteristics of 113 deceased patients with coronavirus disease 2019: retrospective study. BMJ. (2020) 368:m1091. 10.1136/bmj.m1091 32217556 PMC7190011

[80] Xu F Wen Y Hu X Wang T Chen G. The potential use of vitamin C to prevent kidney injury in patients with COVID-19. Diseases. (2021) 9:30046. 10.3390/diseases9030046 34203409 PMC8293113

[81] Martellucci CA Sah R Rabaan AA Dhama K Casalone C Arteaga-Livias K . Changes in the spatial distribution of COVID-19 incidence in Italy using GIS-based maps. Ann Clin Microbiol Antimicrob. (2020) 19:30. 10.1186/s12941-020-00373-z 32682420 PMC7368601

[82] Cases D. Incidence - United States, February 12-April 7, 2020. Morbid Weekly Rep. (2020) 69:465–71. 10.15585/mmwr.mm6915e4 32298250 PMC7755058

[83] Udomkarnjananun S Kerr SJ Townamchai N Susantitaphong P Tulvatana W Praditpornsilpa K . Mortality risk factors of COVID-19 infection in kidney transplantation recipients: a systematic review and meta-analysis of cohorts and clinical registries. Sci Rep. (2021) 11:20073. 10.1038/s41598-021-99713-y 34625642 PMC8501014

[84] Phannajit J Takkavatakarn K Katavetin P Asawavichienjinda T Tungsanga K Praditpornsilpa K . Factors associated with the incidence and mortality of coronavirus disease 2019 (COVID-19) after 126-million cases: a meta-analysis. J Epidemiol Glob Health. (2021) 11:289–95. 10.2991/jegh.k.210527.001 34270185 PMC8435869

[85] Chen JJ Lee TH Tian YC Lee CC Fan PC Chang CH. Immunogenicity rates after SARS-CoV-2 vaccination in people with end-stage kidney disease: a systematic review and meta-analysis. J Am Med Assoc Netw Open. (2021) 4:e2131749. 10.1001/jamanetworkopen.2021.31749 34709385 PMC8554642

