# Supplementary material for: Kidney health in the COVID-19 pandemic: An umbrella review of meta-analyses and systematic reviews
Source: Front Public Health. 2022 Sep 12;10:963667. doi: 10.3389/fpubh.2022.963667 (PMC9511113; doi:10.3389/fpubh.2022.963667)
Supplement: Supplementary file 1 [file Data_Sheet_1.docx]

Supplementary Table S1. The list of 103 reviews included for methodological assessment

1. Fu EL, Janse RJ, de Jong Y, et al. Acute kidney injury and kidney replacement therapy in COVID-19: a systematic review and meta-analysis. *Clinical kidney journal* 2020; **13**(4): 550-63.

2. Ali H, Daoud A, Mohamed MM, et al. Survival rate in acute kidney injury superimposed COVID-19 patients: a systematic review and meta-analysis. *Renal failure* 2020; **42**(1): 393-7.

3. Alves VP, Casemiro FG, Araujo BG, et al. Factors Associated with Mortality among Elderly People in the COVID-19 Pandemic (SARS-CoV-2): A Systematic Review and Meta-Analysis. *International journal of environmental research and public health* 2021; **18**(15).

4. Aronoff SC, Hall A, Del Vecchio MT. The Natural History of Severe Acute Respiratory Syndrome Coronavirus 2-Related Multisystem Inflammatory Syndrome in Children: A Systematic Review. *Journal of the Pediatric Infectious Diseases Society* 2020; **9**(6): 746-51.

5. Awortwe C, Cascorbi I. Meta-analysis on outcome-worsening comorbidities of COVID-19 and related potential drug-drug interactions. *Pharmacological research* 2020; **161**: 105250.

6. Aziz H, Lashkari N, Yoon YC, et al. Effects of Coronavirus Disease 2019 on Solid Organ Transplantation. *Transplantation proceedings* 2020; **52**(9): 2642-53.

7. Bajwa H, Riaz Y, Ammar M, Farooq S, Yousaf A. The Dilemma of Renal Involvement in COVID-19: A Systematic Review. *Cureus* 2020; **12**(6): e8632.

8. Baradaran A, Ebrahimzadeh MH, Baradaran A, Kachooei AR. Prevalence of Comorbidities in COVID-19 Patients: A Systematic Review and Meta-Analysis. *The archives of bone and joint surgery* 2020; **8**(Suppl 1): 247-55.

9. Barek MA, Aziz MA, Islam MS. Impact of age, sex, comorbidities and clinical symptoms on the severity of COVID-19 cases: A meta-analysis with 55 studies and 10014 cases. *Heliyon* 2020; **6**(12): e05684.

10. Bentivegna M, Hulme C, Ebell MH. Primary Care Relevant Risk Factors for Adverse Outcomes in Patients With COVID-19 Infection: A Systematic Review. *Journal of the American Board of Family Medicine : JABFM* 2021; **34**(Suppl): S113-s26.

11. Brienza N, Puntillo F, Romagnoli S, Tritapepe L. Acute Kidney Injury in Coronavirus Disease 2019 Infected Patients: A Meta-Analytic Study. *Blood purification* 2021; **50**(1): 35-41.

12. Cai R, Zhang J, Zhu Y, Liu L, Liu Y, He Q. Mortality in chronic kidney disease patients with COVID-19: a systematic review and meta-analysis. *International urology and nephrology* 2021; **53**(8): 1623-9.

13. Cai X, Wu G, Zhang J, Yang L. Risk Factors for Acute Kidney Injury in Adult Patients With COVID-19: A Systematic Review and Meta-Analysis. *Frontiers in medicine* 2021; **8**: 719472.

14. Chan KW, Yu KY, Lee PW, Lai KN, Tang SC. Global REnal Involvement of CORonavirus Disease 2019 (RECORD): A Systematic Review and Meta-Analysis of Incidence, Risk Factors, and Clinical Outcomes. *Frontiers in medicine* 2021; **8**: 678200.

15. Chan VW, Chiu PK, Yee CH, Yuan Y, Ng CF, Teoh JY. A systematic review on COVID-19: urological manifestations, viral RNA detection and special considerations in urological conditions. *World journal of urology* 2020: 1-12.

16. Chang R, Elhusseiny KM, Yeh YC, Sun WZ. COVID-19 ICU and mechanical ventilation patient characteristics and outcomes-A systematic review and meta-analysis. *PloS one* 2021; **16**(2): e0246318.

17. Cheruiyot I, Henry B, Lippi G, et al. Acute Kidney Injury is Associated with Worse Prognosis In COVID-19 Patients: A Systematic Review and Meta-analysis. *Acta bio-medica : Atenei Parmensis* 2020; **91**(3): e2020029.

18. Chung EY, Palmer SC, Natale P, et al. Incidence and Outcomes of COVID-19 in People With CKD: A Systematic Review and Meta-analysis. *American journal of kidney diseases : the official journal of the National Kidney Foundation* 2021.

19. Dessie ZG, Zewotir T. Mortality-related risk factors of COVID-19: a systematic review and meta-analysis of 42 studies and 423,117 patients. *BMC infectious diseases* 2021; **21**(1): 855.

20. Dorjee K, Kim H, Bonomo E, Dolma R. Prevalence and predictors of death and severe disease in patients hospitalized due to COVID-19: A comprehensive systematic review and meta-analysis of 77 studies and 38,000 patients. *PloS one* 2020; **15**(12): e0243191.

21. Du P, Li D, Wang A, Shen S, Ma Z, Li X. A Systematic Review and Meta-Analysis of Risk Factors Associated with Severity and Death in COVID-19 Patients. *The Canadian journal of infectious diseases & medical microbiology = Journal canadien des maladies infectieuses et de la microbiologie medicale* 2021; **2021**: 6660930.

22. Emami A, Javanmardi F, Pirbonyeh N, Akbari A. Prevalence of Underlying Diseases in Hospitalized Patients with COVID-19: a Systematic Review and Meta-Analysis. *Archives of academic emergency medicine* 2020; **8**(1): e35.

23. Fabrizi F, Alfieri CM, Cerutti R, Lunghi G, Messa P. COVID-19 and Acute Kidney Injury: A Systematic Review and Meta-Analysis. *Pathogens (Basel, Switzerland)* 2020; **9**(12).

24. Fang X, Li S, Yu H, et al. Epidemiological, comorbidity factors with severity and prognosis of COVID-19: a systematic review and meta-analysis. *Aging* 2020; **12**(13): 12493-503.

25. Fathi M, Vakili K, Sayehmiri F, et al. The prognostic value of comorbidity for the severity of COVID-19: A systematic review and meta-analysis study. *PloS one* 2021; **16**(2): e0246190.

26. Fernández Villalobos NV, Ott JJ, Klett-Tammen CJ, et al. Effect modification of the association between comorbidities and severe course of COVID-19 disease by age of study participants: a systematic review and meta-analysis. *Systematic reviews* 2021; **10**(1): 194.

27. Figliozzi S, Masci PG, Ahmadi N, et al. Predictors of adverse prognosis in COVID-19: A systematic review and meta-analysis. *European journal of clinical investigation* 2020; **50**(10): e13362.

28. Hansrivijit P, Qian C, Boonpheng B, et al. Incidence of acute kidney injury and its association with mortality in patients with COVID-19: a meta-analysis. *Journal of investigative medicine : the official publication of the American Federation for Clinical Research* 2020; **68**(7): 1261-70.

29. Ho QY, Sultana R, Lee TL, Thangaraju S, Kee T, Htay H. Coronavirus disease 2019 in kidney transplant recipients: a systematic review and meta-analysis. *Singapore medical journal* 2021.

30. Hoang T, Tran Thi Anh T. Comparison of Comorbidities in Relation to Critical Conditions among Coronavirus Disease 2019 Patients: A Network Meta-Analysis. *Infection & chemotherapy* 2021; **53**(1): 13-28.

31. Izcovich A, Ragusa MA, Tortosa F, et al. Prognostic factors for severity and mortality in patients infected with COVID-19: A systematic review. *PloS one* 2020; **15**(11): e0241955.

32. Jayant K, Reccia I, Bachul PJ, et al. The Impact of COVID-19 on Kidney Transplant Recipients in Pre-Vaccination and Delta Strain Era: A Systematic Review and Meta-Analysis. *Journal of clinical medicine* 2021; **10**(19).

33. Khan MMA, Khan MN, Mustagir MG, Rana J, Islam MS, Kabir MI. Effects of underlying morbidities on the occurrence of deaths in COVID-19 patients: A systematic review and meta-analysis. *Journal of global health* 2020; **10**(2): 020503.

34. Khateri S, Mohammadi H, Khateri R, Moradi Y. The Prevalence of Underlying Diseases and Comorbidities in COVID-19 Patients; an Updated Systematic Review and Meta-analysis. *Archives of academic emergency medicine* 2020; **8**(1): e72.

35. Kremer D, Pieters TT, Verhaar MC, et al. A systematic review and meta-analysis of COVID-19 in kidney transplant recipients: Lessons to be learned. *American journal of transplantation : official journal of the American Society of Transplantation and the American Society of Transplant Surgeons* 2021.

36. Kunutsor SK, Laukkanen JA. Renal complications in COVID-19: a systematic review and meta-analysis. *Annals of medicine* 2020; **52**(7): 345-53.

37. Lee AC, Li WT, Apostol L, et al. Cardiovascular, cerebrovascular, and renal co-morbidities in COVID-19 patients: A systematic-review and meta-analysis. *Computational and structural biotechnology journal* 2021; **19**: 3755-64.

38. Lee SA, Park R, Yang JH, et al. Increased risk of acute kidney injury in coronavirus disease patients with renin-angiotensin-aldosterone-system blockade use: a systematic review and meta-analysis. *Scientific reports* 2021; **11**(1): 13588.

39. Li X, Zhong X, Wang Y, Zeng X, Luo T, Liu Q. Clinical determinants of the severity of COVID-19: A systematic review and meta-analysis. *PloS one* 2021; **16**(5): e0250602.

40. Li Y, Ashcroft T, Chung A, et al. Risk factors for poor outcomes in hospitalised COVID-19 patients: A systematic review and meta-analysis. *Journal of global health* 2021; **11**: 10001.

41. Liang M, Luo N, Chen M, et al. Prevalence and Mortality due to COVID-19 in HIV Co-Infected Population: A Systematic Review and Meta-Analysis. *Infectious diseases and therapy* 2021; **10**(3): 1267-85.

42. Lim MA, Pranata R, Huang I, Yonas E, Soeroto AY, Supriyadi R. Multiorgan Failure With Emphasis on Acute Kidney Injury and Severity of COVID-19: Systematic Review and Meta-Analysis. *Canadian journal of kidney health and disease* 2020; **7**: 2054358120938573.

43. Lin L, Wang X, Ren J, et al. Risk factors and prognosis for COVID-19-induced acute kidney injury: a meta-analysis. *BMJ open* 2020; **10**(11): e042573.

44. Lin YC, Lai TS, Lin SL, Chen YM, Chu TS, Tu YK. Outcomes of coronavirus 2019 infection in patients with chronic kidney disease: a systematic review and meta-analysis. *Therapeutic advances in chronic disease* 2021; **12**: 2040622321998860.

45. Liu X, Li X, Sun T, et al. East-West differences in clinical manifestations of COVID-19 patients: A systematic literature review and meta-analysis. *Journal of medical virology* 2021; **93**(5): 2683-93.

46. Liu YF, Zhang Z, Pan XL, et al. The chronic kidney disease and acute kidney injury involvement in COVID-19 pandemic: A systematic review and meta-analysis. *PloS one* 2021; **16**(1): e0244779.

47. Luo L, Fu M, Li Y, et al. The potential association between common comorbidities and severity and mortality of coronavirus disease 2019: A pooled analysis. *Clinical cardiology* 2020; **43**(12): 1478-93.

48. Marinaki S, Tsiakas S, Korogiannou M, Grigorakos K, Papalois V, Boletis I. A Systematic Review of COVID-19 Infection in Kidney Transplant Recipients: A Universal Effort to Preserve Patients' Lives and Allografts. *Journal of clinical medicine* 2020; **9**(9).

49. Menon T, Gandhi SAQ, Tariq W, et al. Impact of Chronic Kidney Disease on Severity and Mortality in COVID-19 Patients: A Systematic Review and Meta-analysis. *Cureus* 2021; **13**(4): e14279.

50. Menon T, Sharma R, Kataria S, et al. The Association of Acute Kidney Injury With Disease Severity and Mortality in COVID-19: A Systematic Review and Meta-Analysis. *Cureus* 2021; **13**(3): e13894.

51. Mesas AE, Cavero-Redondo I, Álvarez-Bueno C, et al. Predictors of in-hospital COVID-19 mortality: A comprehensive systematic review and meta-analysis exploring differences by age, sex and health conditions. *PloS one* 2020; **15**(11): e0241742.

52. Mirjalili H, Dastgheib SA, Shaker SH, et al. Proportion and mortality of Iranian diabetes mellitus, chronic kidney disease, hypertension and cardiovascular disease patients with COVID-19: a meta-analysis. *Journal of diabetes and metabolic disorders* 2021; **20**(1): 1-13.

53. Mishra P, Parveen R, Bajpai R, Samim M, Agarwal NB. Impact of cardiovascular diseases on severity of COVID-19 patients: A systematic review. *Annals of the Academy of Medicine, Singapore* 2021; **50**(1): 52-60.

54. Nandy K, Salunke A, Pathak SK, et al. Coronavirus disease (COVID-19): A systematic review and meta-analysis to evaluate the impact of various comorbidities on serious events. *Diabetes & metabolic syndrome* 2020; **14**(5): 1017-25.

55. Ng WH, Tipih T, Makoah NA, et al. Comorbidities in SARS-CoV-2 Patients: a Systematic Review and Meta-Analysis. *mBio* 2021; **12**(1).

56. Noor FM, Islam MM. Prevalence and Associated Risk Factors of Mortality Among COVID-19 Patients: A Meta-Analysis. *Journal of community health* 2020; **45**(6): 1270-82.

57. Nopsopon T, Kittrakulrat J, Takkavatakarn K, Eiamsitrakoon T, Kanjanabuch T, Pongpirul K. Covid-19 in end-stage renal disease patients with renal replacement therapies: A systematic review and meta-analysis. *PLoS neglected tropical diseases* 2021; **15**(6): e0009156.

58. Oliveira CB, Lima CAD, Vajgel G, Campos Coelho AV, Sandrin-Garcia P. High burden of acute kidney injury in COVID-19 pandemic: systematic review and meta-analysis. *Journal of clinical pathology* 2020.

59. Oltean M, Søfteland JM, Bagge J, et al. Covid-19 in kidney transplant recipients: a systematic review of the case series available three months into the pandemic. *Infectious diseases (London, England)* 2020; **52**(11): 830-7.

60. Ouyang L, Gong Y, Zhu Y, Gong J. Association of acute kidney injury with the severity and mortality of SARS-CoV-2 infection: A meta-analysis. *The American journal of emergency medicine* 2021; **43**: 149-57.

61. Papadopoulos VP, Koutroulos MV, Zikoudi DG, et al. Diabetes-related acute metabolic emergencies in COVID-19 patients: a systematic review and meta-analysis. *Diabetology international* 2021: 1-15.

62. Phanish M, Ster IC, Ghazanfar A, et al. Systematic Review and Meta-analysis of COVID-19 and Kidney Transplant Recipients, the South West London Kidney Transplant Network Experience. *Kidney international reports* 2021; **6**(3): 574-85.

63. Potere N, Valeriani E, Candeloro M, et al. Acute complications and mortality in hospitalized patients with coronavirus disease 2019: a systematic review and meta-analysis. *Critical care (London, England)* 2020; **24**(1): 389.

64. Pranata R, Huang I, Lawrensia S, et al. Proton pump inhibitor on susceptibility to COVID-19 and its severity: a systematic review and meta-analysis. *Pharmacological reports : PR* 2021: 1-8.

65. Pranata R, Lim MA, Yonas E, et al. Thrombocytopenia as a prognostic marker in COVID-19 patients: diagnostic test accuracy meta-analysis. *Epidemiology and infection* 2021; **149**: e40.

66. Pranata R, Permana H, Huang I, et al. The use of renin angiotensin system inhibitor on mortality in patients with coronavirus disease 2019 (COVID-19): A systematic review and meta-analysis. *Diabetes & metabolic syndrome* 2020; **14**(5): 983-90.

67. Pranata R, Supriyadi R, Huang I, et al. The Association Between Chronic Kidney Disease and New Onset Renal Replacement Therapy on the Outcome of COVID-19 Patients: A Meta-analysis. *Clinical medicine insights Circulatory, respiratory and pulmonary medicine* 2020; **14**: 1179548420959165.

68. Qian Z, Lu S, Luo X, Chen Y, Liu L. Mortality and Clinical Interventions in Critically ill Patient With Coronavirus Disease 2019: A Systematic Review and Meta-Analysis. *Frontiers in medicine* 2021; **8**: 635560.

69. Raina R, Chakraborty R, Mawby I, Agarwal N, Sethi S, Forbes M. Critical analysis of acute kidney injury in pediatric COVID-19 patients in the intensive care unit. *Pediatric nephrology (Berlin, Germany)* 2021; **36**(9): 2627-38.

70. Raina R, Mahajan ZA, Vasistha P, et al. Incidence and Outcomes of Acute Kidney Injury in COVID-19: A Systematic Review. *Blood purification* 2021: 1-14.

71. Raja MA, Mendoza MA, Villavicencio A, et al. COVID-19 in solid organ transplant recipients: A systematic review and meta-analysis of current literature. *Transplantation reviews (Orlando, Fla)* 2021; **35**(1): 100588.

72. Robbins-Juarez SY, Qian L, King KL, et al. Outcomes for Patients With COVID-19 and Acute Kidney Injury: A Systematic Review and Meta-Analysis. *Kidney international reports* 2020; **5**(8): 1149-60.

73. Sahu AK, Mathew R, Aggarwal P, et al. Clinical Determinants of Severe COVID-19 Disease - A Systematic Review and Meta-Analysis. *Journal of global infectious diseases* 2021; **13**(1): 13-9.

74. Schlesinger S, Neuenschwander M, Lang A, et al. Risk phenotypes of diabetes and association with COVID-19 severity and death: a living systematic review and meta-analysis. *Diabetologia* 2021; **64**(7): 1480-91.

75. Shao M, Li X, Liu F, Tian T, Luo J, Yang Y. Acute kidney injury is associated with severe infection and fatality in patients with COVID-19: A systematic review and meta-analysis of 40 studies and 24,527 patients. *Pharmacological research* 2020; **161**: 105107.

76. Shi C, Wang L, Ye J, et al. Predictors of mortality in patients with coronavirus disease 2019: a systematic review and meta-analysis. *BMC infectious diseases* 2021; **21**(1): 663.

77. Shi Q, Wang Z, Liu J, et al. Risk factors for poor prognosis in children and adolescents with COVID-19: A systematic review and meta-analysis. *EClinicalMedicine* 2021; **41**: 101155.

78. Silver SA, Beaubien-Souligny W, Shah PS, et al. The Prevalence of Acute Kidney Injury in Patients Hospitalized With COVID-19 Infection: A Systematic Review and Meta-analysis. *Kidney medicine* 2021; **3**(1): 83-98.e1.

79. Singh AK, Gillies CL, Singh R, et al. Prevalence of co-morbidities and their association with mortality in patients with COVID-19: A systematic review and meta-analysis. *Diabetes, obesity & metabolism* 2020; **22**(10): 1915-24.

80. Singh J, Malik P, Patel N, et al. Kidney disease and COVID-19 disease severity-systematic review and meta-analysis. *Clinical and experimental medicine* 2021: 1-11.

81. Ssentongo P, Ssentongo AE, Heilbrunn ES, Ba DM, Chinchilli VM. Association of cardiovascular disease and 10 other pre-existing comorbidities with COVID-19 mortality: A systematic review and meta-analysis. *PloS one* 2020; **15**(8): e0238215.

82. Subramaniam A, Lim ZJ, Ponnapa Reddy M, Shekar K. A systematic review and meta-analysis of the characteristics and outcomes of readmitted COVID-19 survivors. *Internal medicine journal* 2021.

83. Taylor EH, Marson EJ, Elhadi M, et al. Factors associated with mortality in patients with COVID-19 admitted to intensive care: a systematic review and meta-analysis. *Anaesthesia* 2021; **76**(9): 1224-32.

84. Thakur B, Dubey P, Benitez J, et al. A systematic review and meta-analysis of geographic differences in comorbidities and associated severity and mortality among individuals with COVID-19. *Scientific reports* 2021; **11**(1): 8562.

85. Tiruneh SA, Tesema ZT, Azanaw MM, Angaw DA. The effect of age on the incidence of COVID-19 complications: a systematic review and meta-analysis. *Systematic reviews* 2021; **10**(1): 80.

86. Toraih EA, Hussein MH, Elshazli RM, et al. Multisystem inflammatory syndrome in pediatric COVID-19 patients: a meta-analysis. *World journal of pediatrics : WJP* 2021; **17**(2): 141-51.

87. Wang B, Li R, Lu Z, Huang Y. Does comorbidity increase the risk of patients with COVID-19: evidence from meta-analysis. *Aging* 2020; **12**(7): 6049-57.

88. Wang B, Luo Q, Zhang W, et al. The Involvement of Chronic Kidney Disease and Acute Kidney Injury in Disease Severity and Mortality in Patients with COVID-19: A Meta-Analysis. *Kidney & blood pressure research* 2021; **46**(1): 17-30.

89. Wang Z, Deng H, Ou C, et al. Clinical symptoms, comorbidities and complications in severe and non-severe patients with COVID-19: A systematic review and meta-analysis without cases duplication. *Medicine* 2020; **99**(48): e23327.

90. Wu T, Zuo Z, Kang S, et al. Multi-organ Dysfunction in Patients with COVID-19: A Systematic Review and Meta-analysis. *Aging and disease* 2020; **11**(4): 874-94.

91. Wu YY, Li HY, Xu XB, Zheng KX, Qi XS, Guo XZ. [Clinical features and outcome of treatment for novel coronavirus pneumonia: a meta-analysis]. *Zhonghua gan zang bing za zhi = Zhonghua ganzangbing zazhi = Chinese journal of hepatology* 2020; **28**(3): 240-6.

92. Xiang G, Xie L, Chen Z, et al. Clinical risk factors for mortality of hospitalized patients with COVID-19: systematic review and meta-analysis. *Annals of palliative medicine* 2021; **10**(3): 2723-35.

93. Xu Z, Tang Y, Huang Q, et al. Systematic review and subgroup analysis of the incidence of acute kidney injury (AKI) in patients with COVID-19. *BMC nephrology* 2021; **22**(1): 52.

94. Yang Q, Yang X. Incidence and risk factors of kidney impairment on patients with COVID-19: A meta-analysis of 10180 patients. *PloS one* 2020; **15**(11): e0241953.

95. Yang X, Jin Y, Li R, Zhang Z, Sun R, Chen D. Prevalence and impact of acute renal impairment on COVID-19: a systematic review and meta-analysis. *Critical care (London, England)* 2020; **24**(1): 356.

96. Yin T, Li Y, Ying Y, Luo Z. Prevalence of comorbidity in Chinese patients with COVID-19: systematic review and meta-analysis of risk factors. *BMC infectious diseases* 2021; **21**(1): 200.

97. Zhang L, Hou J, Ma FZ, Li J, Xue S, Xu ZG. The common risk factors for progression and mortality in COVID-19 patients: a meta-analysis. *Archives of virology* 2021; **166**(8): 2071-87.

98. Zhang T, Huang WS, Guan W, et al. Risk factors and predictors associated with the severity of COVID-19 in China: a systematic review, meta-analysis, and meta-regression. *Journal of thoracic disease* 2020; **12**(12): 7429-41.

99. Zhong Z, Li H, Zhu J, et al. Clinical characteristics of 2,459 severe or critically ill COVID-19 patients: A meta-analysis. *Medicine* 2021; **100**(5): e23781.

100. Zhou S, Xu J, Xue C, Yang B, Mao Z, Ong ACM. Coronavirus-associated kidney outcomes in COVID-19, SARS, and MERS: a meta-analysis and systematic review. *Renal failure* 2020; **43**(1): 1-15.

101. Zhou Y, Ren Q, Chen G, et al. Chronic Kidney Diseases and Acute Kidney Injury in Patients With COVID-19: Evidence From a Meta-Analysis. *Frontiers in medicine* 2020; **7**: 588301.

102. Zhu J, Ji P, Pang J, et al. Clinical characteristics of 3062 COVID-19 patients: A meta-analysis. *Journal of medical virology* 2020; **92**(10): 1902-14.

103. Zinellu A, Mangoni AA. Cystatin C, COVID-19 severity and mortality: a systematic review and meta-analysis. *Journal of nephrology* 2021: 1-10.

Supplementary Table S2. Methodological quality assessment using ROBIS tool

|  | Phase 1 | Phase 2 |  |  |  |  |  |  |  |  |  |  |  |  |  |  |  |  |  |  |  |  |  |  |  |  |  | Phase 3 |  |  |  |
| --- | --- | --- | --- | --- | --- | --- | --- | --- | --- | --- | --- | --- | --- | --- | --- | --- | --- | --- | --- | --- | --- | --- | --- | --- | --- | --- | --- | --- | --- | --- | --- |
|  |  | DOMAIN1 | |  |  |  |  | DOMAIN2 | |  |  |  |  | DOMAIN3 | |  |  |  |  |  | DOMAIN4 | |  |  |  |  |  | DOMAIN1 | DOMAIN2 | DOMAIN3 | risk of bias |
|  |  | 1.1 | 1.2 | 1.3 | 1.4 | 1.5 | concern | 2.1 | 2.2 | 2.3 | 2.4 | 2.5 | concern | 3.1 | 3.2 | 3.3 | 3.4 | 3.5 |  | concern | 4.1 | 4.2 | 4.3 | 4.4 | 4.5 | 4.6 | concern |  |  |  |  |
| Fu, E. L., 2020 | Y | PY | Y | Y | PY | Y | low | Y | Y | PY | PY | Y | low | Y | Y | Y | Y | PY |  | low | Y | PY | Y | PY | Y | Y | low | Y | Y | Y | low |
| Cai, X., 2021 | Y | Y | Y | PY | PY | Y | low | Y | Y | PY | PY | Y | low | Y | Y | Y | Y | PY |  | low | Y | PY | Y | PN | Y | Y | low | Y | Y | Y | low |
| Chan, K. W., 2021 | Y | Y | Y | Y | PY | Y | low | Y | Y | PY | PY | Y | low | Y | Y | Y | Y | PY |  | low | Y | Y | Y | PY | Y | Y | low | Y | Y | Y | low |
| Chang, R., 2021 | Y | PY | Y | Y | PY | Y | low | Y | Y | PY | PY | Y | low | Y | Y | Y | Y | PY |  | low | Y | Y | Y | PY | Y | Y | low | Y | Y | Y | low |
| Chung, E. Y., 2021 | Y | Y | Y | Y | PY | Y | low | Y | Y | PY | PY | Y | low | Y | Y | Y | Y | PY |  | low | Y | Y | Y | PY | Y | Y | low | Y | Y | Y | low |
| Dessie, Z. G., 2021 | Y | PY | Y | Y | PY | Y | low | Y | Y | PY | PY | Y | low | Y | Y | Y | Y | PY |  | low | Y | Y | Y | PY | Y | Y | low | Y | Y | Y | low |
| Du, P., 2021 | Y | Y | Y | Y | PY | Y | low | Y | Y | PY | PY | Y | low | Y | Y | Y | Y | PY |  | low | Y | Y | Y | PN | Y | Y | low | Y | Y | Y | low |
| Hansrivijit, P., 2020 | Y | Y | Y | Y | PY | Y | low | Y | Y | PY | PY | Y | low | Y | Y | Y | Y | PY |  | low | Y | Y | Y | PY | PY | Y | low | Y | Y | Y | low |
| Ho, Q. Y., 2021 | Y | Y | Y | Y | PY | Y | low | Y | Y | PY | PY | Y | low | Y | Y | Y | Y | PY |  | low | Y | PY | Y | PN | Y | Y | low | Y | Y | Y | low |
| Izcovich, A.,2020 | Y | PY | Y | PY | PY | Y | low | Y | Y | PY | PY | Y | low | Y | Y | Y | Y | PY |  | low | Y | PY | Y | PN | Y | Y | low | Y | Y | Y | low |
| Kremer, D., 2021 | Y | Y | Y | Y | PY | Y | low | Y | Y | PY | PY | Y | low | Y | Y | Y | Y | PY |  | low | Y | Y | Y | PY | Y | Y | low | Y | Y | Y | low |
| Lee, A. C., 2021 | Y | Y | Y | Y | PY | Y | low | Y | Y | PY | PY | Y | low | Y | Y | Y | Y | PY |  | low | Y | Y | Y | PN | Y | Y | low | Y | Y | Y | low |
| Li, Y., 2021 | Y | Y | Y | Y | PY | Y | low | Y | Y | PY | PY | Y | low | Y | Y | Y | Y | PY |  | low | Y | PY | Y | PY | Y | Y | low | Y | Y | Y | low |
| Lim, M. A., 2020 | Y | PY | Y | Y | PY | Y | low | Y | Y | PY | PY | Y | low | Y | Y | Y | Y | PY |  | low | Y | PY | Y | PY | Y | Y | low | Y | Y | Y | low |
| Liu, Y. F., 2021 | Y | Y | Y | Y | PY | Y | low | Y | Y | PY | PY | Y | low | Y | Y | Y | Y | PY |  | low | Y | PY | Y | PN | Y | Y | low | Y | Y | Y | low |
| Luo, L., 2020 | Y | Y | Y | Y | PY | Y | low | Y | Y | PY | PY | Y | low | Y | Y | Y | Y | PY |  | low | Y | Y | Y | PY | Y | Y | low | Y | Y | Y | low |
| Menon, T., 2021 (1) | Y | Y | Y | Y | PY | Y | low | Y | Y | PY | PY | Y | low | Y | Y | Y | Y | PY |  | low | Y | Y | Y | PN | Y | Y | low | Y | Y | Y | low |
| Mesas, A. E., 2020 | Y | Y | Y | Y | PY | Y | low | Y | Y | PY | PY | Y | low | Y | Y | Y | Y | PY |  | low | Y | PY | Y | PY | Y | Y | low | Y | Y | Y | low |
| Mirjalili, H., 2021 | Y | PY | Y | Y | PY | Y | low | Y | Y | PY | PY | Y | low | Y | Y | Y | Y | PY |  | low | Y | Y | Y | PN | Y | Y | low | Y | Y | Y | low |
| Oltean, M., 2020 | Y | PY | Y | Y | PY | Y | low | Y | Y | PY | PY | Y | low | Y | Y | Y | Y | PY |  | low | Y | Y | Y | PY | Y | Y | low | Y | Y | Y | low |
| Papadopoulos, V. P., 2021 | Y | PY | Y | Y | PY | Y | low | Y | Y | PY | PY | Y | low | Y | Y | Y | Y | PY |  | low | Y | Y | Y | PN | Y | Y | low | Y | Y | Y | low |
| Schlesinger, S., 2021 | Y | Y | Y | Y | PY | Y | low | Y | Y | PY | PY | Y | low | Y | Y | Y | Y | PY |  | low | Y | Y | Y | PN | Y | Y | low | Y | Y | Y | low |
| Shi, Q., 2021 | Y | Y | Y | PY | PY | Y | low | Y | Y | PY | PY | Y | low | Y | Y | Y | Y | PY |  | low | Y | PY | Y | PN | Y | Y | low | Y | Y | Y | low |
| Ssentongo, P., 2020 | Y | Y | Y | Y | PY | Y | low | Y | Y | PY | PY | Y | low | Y | Y | Y | Y | PY |  | low | Y | Y | Y | PY | Y | Y | low | Y | Y | Y | low |
| Taylor, E. H., 2021 | Y | Y | Y | Y | PY | Y | low | Y | Y | PY | PY | Y | low | Y | Y | Y | Y | PY |  | low | Y | Y | Y | PY | Y | Y | low | Y | Y | Y | low |
| Wang, B., 2020 | Y | Y | Y | PY | PY | Y | low | Y | Y | PY | PY | Y | low | Y | Y | Y | Y | PY |  | low | Y | Y | Y | PN | Y | Y | low | Y | Y | Y | low |
| Zhang, L., 2021 | Y | Y | Y | Y | PY | Y | low | Y | Y | PY | PY | Y | low | Y | Y | Y | Y | PY |  | low | Y | PY | Y | PY | Y | Y | low | Y | Y | Y | low |
| Zhang, T., 2020 | Y | PY | Y | Y | PY | Y | low | Y | Y | PY | PY | Y | low | Y | Y | Y | Y | PY |  | low | Y | PY | Y | PN | Y | Y | low | Y | Y | Y | low |
| Zhou, S., 2020 | Y | Y | Y | PY | PY | Y | low | Y | Y | PY | PY | Y | low | Y | Y | Y | Y | PY |  | low | Y | PY | Y | PN | Y | Y | low | Y | Y | Y | low |
| Zhou, Y., 2020 | Y | Y | Y | PY | PY | Y | low | Y | Y | PY | PY | Y | low | Y | Y | Y | Y | PY |  | low | Y | PY | Y | PN | Y | Y | low | Y | Y | Y | low |
| Ali, H., 2020 | Y | N | Y | PN | PY | Y | high | Y | Y | PY | PY | Y | low | Y | Y | Y | Y | Y |  | low | Y | N | Y | N | Y | N | high | N | Y | Y | high |
| Alves, V. P., 2020 | Y | N | Y | PY | PY | Y | high | Y | Y | PY | PY | Y | low | Y | Y | Y | Y | Y |  | low | Y | N | Y | N | N | N | high | N | Y | Y | high |
| Aronoff, S. C., 2020 | Y | N | Y | PN | PY | Y | high | Y | Y | PY | PY | Y | low | Y | Y | Y | Y | Y |  | low | Y | N | Y | N | N | N | high | N | Y | Y | high |
| Awortwe, C., 2020 | Y | N | Y | Y | PY | Y | high | Y | Y | PY | PY | Y | low | Y | Y | Y | Y | Y |  | low | Y | N | Y | N | Y | N | high | N | Y | Y | high |
| Aziz, H., 2020 | Y | N | Y | Y | PY | Y | high | Y | Y | PY | PY | Y | low | Y | Y | Y | Y | Y |  | low | Y | N | Y | PN | N | N | high | N | Y | Y | high |
| Bajwa, H., 2020 | Y | N | Y | Y | PY | Y | high | Y | Y | PY | PY | Y | low | Y | Y | Y | Y | Y |  | low | Y | N | Y | N | N | N | high | N | Y | Y | high |
| Baradaran, A., 2020 | Y | N | Y | Y | PY | Y | high | Y | Y | PY | PY | Y | low | Y | Y | Y | Y | Y |  | low | Y | N | Y | N | N | N | high | N | Y | Y | high |
| Barek, M. A., 2020 | Y | N | Y | PY | PY | Y | high | Y | Y | PY | PY | Y | low | Y | Y | Y | Y | Y |  | low | Y | N | Y | PY | Y | N | high | N | Y | Y | high |
| Bentivegna, M., 2021 | Y | Y | Y | Y | PY | Y | low | Y | Y | PY | PY | Y | low | Y | Y | Y | Y | Y |  | low | PY | PY | Y | N | N | N | high | N | Y | Y | high |
| Brienza, N., 2021 | Y | N | Y | Y | PY | Y | high | Y | Y | PY | PY | Y | low | Y | Y | Y | Y | Y |  | low | Y | N | Y | N | N | N | high | N | Y | Y | high |
| Cai, R., 2021 | Y | N | Y | Y | PY | Y | high | Y | Y | PY | PY | Y | low | Y | Y | Y | Y | Y |  | low | Y | N | Y | N | N | N | high | N | Y | Y | high |
| Chan, V. W., 2020 | Y | N | Y | Y | PY | Y | high | Y | Y | PY | PY | Y | low | Y | Y | Y | Y | Y |  | low | Y | N | Y | N | N | N | high | N | Y | Y | high |
| Cheruiyot, I., 2020 | Y | Y | Y | Y | PY | Y | low | Y | Y | PY | PY | Y | low | Y | Y | Y | Y | Y |  | low | Y | PY | Y | N | N | N | high | N | Y | Y | high |
| Dorjee, K., 2020 | Y | N | Y | Y | PY | Y | high | Y | Y | PY | PY | Y | low | Y | Y | Y | Y | Y |  | low | Y | N | Y | N | N | N | high | N | Y | Y | high |
| Emami, A., 2020 | Y | N | Y | Y | PY | Y | high | Y | Y | PY | PY | Y | low | Y | Y | Y | Y | Y |  | low | PY | N | Y | N | Y | N | high | N | Y | Y | high |
| Fabrizi, F., 2020 | Y | N | Y | Y | PY | Y | high | Y | Y | PY | PY | Y | low | Y | Y | Y | Y | Y |  | low | Y | N | Y | N | Y | N | high | N | Y | Y | high |
| Fang, X., 2020 | Y | N | Y | Y | PY | Y | high | Y | Y | PY | PY | Y | low | Y | Y | Y | Y | Y |  | low | Y | N | Y | N | N | N | high | N | Y | Y | high |
| Fathi, M., 2021 | Y | N | Y | Y | PY | Y | high | Y | Y | PY | PY | Y | low | Y | Y | Y | Y | Y |  | low | Y | N | Y | N | Y | N | high | N | Y | Y | high |
| Fernández Villalobos, N. V., 2021 | Y | Y | Y | Y | PY | Y | low | Y | Y | PY | PY | Y | low | Y | Y | PY | Y | Y |  | low | Y | Y | Y | N | N | N | high | N | Y | Y | high |
| Figliozzi, S., 2020 | Y | Y | Y | Y | PY | Y | low | Y | Y | PY | PY | Y | low | Y | Y | PY | Y | Y |  | low | Y | Y | Y | PY | N | N | high | N | Y | Y | high |
| Hoang, T., 2021 | Y | N | Y | Y | PY | Y | high | Y | Y | PY | PY | Y | low | Y | Y | Y | Y | Y |  | low | Y | N | Y | N | Y | N | high | N | Y | Y | high |
| Jayant, K., 2021 | Y | Y | Y | Y | PY | Y | low | Y | Y | PY | PY | Y | low | Y | Y | Y | Y | Y |  | low | Y | Y | Y | N | N | N | high | N | Y | Y | high |
| Khan, M. M. A., 2020 | Y | N | Y | Y | PY | Y | high | Y | Y | PY | PY | Y | low | Y | Y | Y | Y | Y |  | low | Y | N | Y | N | N | N | high | N | Y | Y | high |
| Khateri, S., 2020 | Y | N | Y | Y | PY | Y | high | Y | Y | PY | PY | Y | low | Y | Y | Y | Y | Y |  | low | Y | N | Y | N | N | N | high | N | Y | Y | high |
| Kunutsor, S. K., 2020 | Y | Y | Y | Y | PY | Y | low | Y | Y | PY | PY | Y | low | Y | Y | N | Y | Y |  | low | Y | Y | Y | N | N | N | high | N | Y | Y | high |
| Lee, S. A., 2021 | Y | N | Y | Y | PY | Y | high | Y | Y | PY | PY | Y | low | Y | Y | Y | Y | Y |  | low | Y | N | Y | N | Y | N | high | N | Y | Y | high |
| Li, X., 2021 | Y | N | Y | Y | PY | Y | high | Y | Y | PY | PY | Y | low | Y | Y | Y | Y | Y |  | low | Y | N | Y | N | Y | N | high | N | Y | Y | high |
| Liang, M., 2021 | Y | N | Y | Y | PY | Y | high | Y | Y | PY | PY | Y | low | Y | Y | Y | Y | Y |  | low | Y | N | Y | N | N | N | high | N | Y | Y | high |
| Lin, L., 2020 | Y | N | Y | Y | PY | Y | high | Y | Y | PY | PY | Y | low | Y | Y | Y | Y | Y |  | low | Y | N | Y | PN | Y | N | high | N | Y | Y | high |
| Lin, Y. C., 2021 | Y | Y | Y | Y | PY | Y | low | Y | Y | PY | PY | Y | low | Y | Y | Y | Y | Y |  | low | Y | Y | Y | N | Y | N | high | N | Y | Y | high |
| Liu, X., 2021 | Y | Y | Y | Y | PY | Y | low | Y | Y | PY | PY | Y | low | Y | Y | Y | Y | Y |  | low | Y | PY | Y | N | N | N | high | N | Y | Y | high |
| Marinaki, S., 2020 | Y | N | Y | Y | PY | Y | high | Y | Y | PY | PY | Y | low | Y | Y | Y | Y | Y |  | low | Y | N | Y | N | N | N | high | N | Y | Y | high |
| Menon, T., 2021 (2) | Y | N | Y | Y | PY | Y | high | Y | Y | PY | PY | Y | low | Y | Y | Y | Y | Y |  | low | Y | N | Y | N | N | N | high | N | Y | Y | high |
| Mishra, P., 2021 | Y | N | Y | Y | PY | Y | high | Y | Y | PY | PY | Y | low | Y | Y | Y | Y | Y |  | low | Y | N | Y | N | Y | N | high | N | Y | Y | high |
| Nandy, K., 2020 | Y | N | Y | Y | PY | Y | high | Y | Y | PY | PY | Y | low | Y | Y | Y | Y | Y |  | low | Y | N | Y | N | N | N | high | N | Y | Y | high |
| Ng, W. H., 2021 | Y | N | Y | PN | PY | Y | high | Y | Y | PY | PY | Y | low | Y | Y | Y | Y | Y |  | low | Y | N | Y | N | N | N | high | N | Y | Y | high |
| Noor, F. M., 2020 | Y | N | Y | Y | PY | Y | high | Y | Y | PY | PY | Y | low | Y | Y | Y | Y | Y |  | low | Y | N | Y | N | N | N | high | N | Y | Y | high |
| Nopsopon, T., 2021 | Y | Y | Y | Y | PY | Y | low | Y | Y | PY | PY | Y | low | Y | Y | Y | Y | Y |  | low | Y | Y | Y | N | N | N | high | N | Y | Y | high |
| Oliveira, C. B., 2021 | Y | N | Y | Y | PY | Y | high | Y | Y | PY | PY | Y | low | Y | Y | PN | Y | Y |  | high | Y | N | Y | N | N | N | high | N | Y | Y | high |
| Ouyang, L., 2021 | Y | N | Y | Y | PY | Y | high | Y | Y | PY | PY | Y | low | Y | Y | PN | Y | Y |  | high | Y | N | Y | N | N | N | high | N | Y | Y | high |
| Phanish, M., 2021 | Y | N | Y | Y | PY | Y | high | Y | Y | PY | PY | Y | low | Y | Y | Y | Y | Y |  | low | Y | N | Y | N | N | N | high | N | Y | Y | high |
| Potere, N., 2020 | Y | Y | Y | Y | PY | Y | low | Y | Y | PY | PY | Y | low | Y | Y | Y | Y | Y |  | low | Y | Y | Y | PN | N | N | high | N | Y | Y | high |
| Pranata, R., 2020 (1) | Y | Y | Y | Y | PY | Y | low | Y | Y | PY | PY | Y | low | Y | Y | Y | Y | Y |  | low | Y | Y | Y | N | Y | N | high | N | Y | Y | high |
| Pranata, R., 2020 (2) | Y | Y | Y | Y | PY | Y | low | Y | Y | PY | PY | Y | low | Y | Y | Y | Y | Y |  | low | Y | Y | Y | N | Y | N | high | N | Y | Y | high |
| Pranata, R., 2020 (3) | Y | N | Y | Y | PY | Y | high | Y | Y | PY | PY | Y | low | Y | Y | Y | Y | Y |  | low | Y | N | Y | N | Y | N | high | N | Y | Y | high |
| Pranata, R., 2020 (4) | Y | N | Y | Y | PY | Y | high | Y | Y | PY | PY | Y | low | Y | Y | Y | Y | Y |  | low | Y | N | Y | N | Y | N | high | N | Y | Y | high |
| Qian, Z., 2021 | Y | N | Y | PY | PY | Y | high | Y | Y | PY | PY | Y | low | Y | Y | Y | Y | Y |  | low | Y | N | Y | N | N | N | high | N | Y | Y | high |
| Raina, R., 2021 (1) | Y | Y | Y | PN | PY | Y | low | Y | Y | PY | PY | Y | low | Y | Y | Y | Y | Y |  | low | Y | PY | Y | N | Y | N | high | N | Y | Y | high |
| Raina, R., 2021 (2) | Y | N | Y | PY | PY | Y | high | Y | Y | PY | PY | Y | low | Y | Y | Y | Y | Y |  | low | Y | N | Y | N | Y | N | high | N | Y | Y | high |
| Raja, M. A., 2021 | Y | N | Y | Y | PY | Y | high | Y | N | PY | PY | Y | low | Y | Y | Y | Y | Y |  | low | Y | N | Y | N | N | N | high | N | Y | Y | high |
| Robbins-Juarez, S. Y., 2020 | Y | N | Y | Y | PY | Y | high | Y | Y | PY | PY | Y | low | Y | Y | Y | Y | Y |  | low | Y | N | Y | N | Y | N | high | N | Y | Y | high |
| Sahu, A. K., 2021 | Y | N | Y | Y | PY | Y | high | Y | Y | PY | PY | Y | low | Y | Y | Y | Y | Y |  | low | Y | N | Y | N | Y | N | high | N | Y | Y | high |
| Shao, M., 2020 | Y | N | Y | Y | PY | Y | high | Y | Y | PY | PY | Y | low | Y | Y | Y | Y | Y |  | low | Y | N | Y | N | Y | N | high | N | Y | Y | high |
| Shi, C., 2021 | Y | N | Y | Y | PY | Y | high | Y | Y | PY | PY | Y | low | Y | Y | Y | Y | Y |  | low | Y | N | Y | N | N | N | high | N | Y | Y | high |
| Silver, S. A., 2021 | Y | N | Y | Y | PY | Y | high | Y | Y | PY | PY | Y | low | Y | Y | Y | Y | Y |  | low | Y | N | Y | N | N | N | high | N | Y | Y | high |
| Singh, A. K., 2020 | Y | N | Y | Y | PY | Y | high | Y | Y | PY | PY | Y | low | Y | Y | N | Y | Y |  | high | Y | N | Y | N | N | N | high | N | Y | Y | high |
| Singh, J., 2021 | Y | N | Y | Y | PY | Y | high | Y | Y | PY | PY | Y | low | Y | Y | Y | Y | Y |  | low | Y | N | Y | N | N | N | high | N | Y | Y | high |
| Subramaniam, A., 2021 | Y | N | Y | Y | PY | Y | high | Y | Y | PY | PY | Y | low | Y | Y | Y | Y | Y |  | low | Y | N | Y | N | N | N | high | N | Y | Y | high |
| Thakur, B., 2021 | Y | N | Y | Y | PY | Y | high | Y | Y | PY | PY | Y | low | Y | Y | Y | Y | Y |  | low | Y | N | Y | N | N | N | high | N | Y | Y | high |
| Tiruneh, S. A., 2021 | Y | Y | Y | Y | PY | Y | low | Y | Y | PY | PY | Y | low | Y | Y | Y | Y | Y |  | low | Y | Y | Y | N | Y | N | high | N | Y | Y | high |
| Toraih, E. A., 2021 | Y | N | Y | Y | PY | Y | high | Y | Y | PY | PY | Y | low | Y | Y | Y | Y | Y |  | low | Y | N | Y | N | N | N | high | N | Y | Y | high |
| Wang, B., 2020 | Y | N | Y | Y | PY | Y | high | Y | Y | PY | PY | Y | low | Y | Y | N | Y | Y |  | high | Y | N | Y | PN | Y | N | high | N | Y | Y | high |
| Wang, Z., 2020 | Y | Y | Y | Y | PY | Y | low | Y | Y | PY | PY | Y | low | Y | Y | Y | Y | Y |  | low | Y | Y | Y | N | Y | N | high | N | Y | Y | high |
| Wu, T., 2020 | Y | Y | Y | Y | PY | Y | low | Y | Y | PY | PY | Y | low | Y | Y | Y | Y | Y |  | low | Y | Y | Y | N | N | N | high | N | Y | Y | high |
| Wu, Y. Y., 2020 | Y | N | Y | Y | PY | Y | high | Y | Y | PY | PY | Y | low | Y | Y | Y | Y | Y |  | low | Y | N | Y | N | N | N | high | N | Y | Y | high |
| Xiang, G., 2021 | Y | N | Y | Y | PY | Y | high | Y | Y | PY | PY | Y | low | Y | Y | Y | Y | Y |  | low | Y | N | Y | N | Y | N | high | N | Y | Y | high |
| Xu, Z., 2021 | Y | N | Y | Y | PY | Y | high | Y | Y | PY | PY | Y | low | Y | Y | Y | Y | Y |  | low | Y | N | Y | N | N | N | high | N | Y | Y | high |
| Yang, Q., 2020 | Y | N | Y | Y | PY | Y | high | Y | N | PY | PY | Y | low | Y | Y | Y | Y | Y |  | low | Y | N | Y | N | Y | N | high | N | Y | Y | high |
| Yang, X., 2020 | Y | N | Y | Y | PY | Y | high | Y | Y | PY | PY | Y | low | Y | Y | Y | Y | Y |  | low | Y | N | Y | N | N | N | high | N | Y | Y | high |
| Yin, T., 2021 | Y | Y | Y | Y | PY | Y | low | Y | Y | PY | PY | Y | low | Y | Y | N | Y | Y |  | high | Y | Y | Y | N | N | N | high | N | Y | Y | high |
| Zhong, Z., 2021 | Y | N | Y | Y | PY | Y | high | Y | Y | PY | PY | Y | low | Y | Y | Y | Y | Y |  | low | Y | N | Y | N | Y | N | high | N | Y | Y | high |
| Zhu, J., 2020 | Y | N | Y | Y | PY | Y | high | Y | Y | PY | PY | Y | low | Y | Y | Y | Y | Y |  | low | Y | N | Y | N | Y | N | high | N | Y | Y | high |
| Zinellu, A., 2021 | Y | Y | Y | Y | PY | Y | low | Y | Y | PY | PY | Y | low | Y | Y | Y | Y | Y |  | low | Y | Y | Y | N | Y | N | high | N | Y | Y | high |

Supplementary Table S3. The list of 119 primary studies incorporated for review update

1. Abdallah E, Al Helal B, Asad R, et al. Incidence and Outcomes of Acute Kidney Injury in Critically Ill Patients with Coronavirus Disease 2019. *Saudi journal of kidney diseases and transplantation : an official publication of the Saudi Center for Organ Transplantation, Saudi Arabia* 2021;32(1):84-91. doi: 10.4103/1319-2442.318551 [published Online First: 2021/06/20]

2. AbdelGhaffar MM, Omran D, Elgebaly A, et al. Prediction of mortality in hospitalized Egyptian patients with Coronavirus disease-2019: A multicenter retrospective study. *PloS one* 2022;17(1):e0262348. doi: 10.1371/journal.pone.0262348 [published Online First: 2022/01/12]

3. Ahmed W, Al Obaidli AAK, Joseph P, et al. Outcomes of patients with end stage kidney disease on dialysis with COVID-19 in Abu Dhabi, United Arab Emirates; from PCR to antibody. *BMC nephrology* 2021;22(1):198. doi: 10.1186/s12882-021-02378-y [published Online First: 2021/05/28]

4. Alessandri F, Pistolesi V, Manganelli C, et al. Acute Kidney Injury and COVID-19: A Picture from an Intensive Care Unit. *Blood purification* 2021;50(6):767-71. doi: 10.1159/000513153 [published Online First: 2021/01/08]

5. Alkadi MM, Al-Malki HA, Asim M, et al. Kidney Transplant Recipients Infected With Coronavirus Disease 2019: Retrospective Qatar Experience. *Transplantation proceedings* 2021;53(8):2438-46. doi: 10.1016/j.transproceed.2021.06.001 [published Online First: 2021/07/20]

6. AlOtaibi TM, Gheith OA, Abuelmagd MM, et al. Better outcome of COVID-19 positive kidney transplant recipients during the unremitting stage with optimized anticoagulation and immunosuppression. *Clinical transplantation* 2021;35(6):e14297. doi: 10.1111/ctr.14297 [published Online First: 2021/03/27]

7. Arikan H, Ozturk S, Tokgoz B, et al. Characteristics and outcomes of acute kidney injury in hospitalized COVID-19 patients: A multicenter study by the Turkish society of nephrology. *PloS one* 2021;16(8):e0256023. doi: 10.1371/journal.pone.0256023 [published Online First: 2021/08/11]

8. Bajpai D, Deb S, Bose S, et al. Recovery of kidney function after AKI because of COVID-19 in kidney transplant recipients. *Transplant international : official journal of the European Society for Organ Transplantation* 2021;34(6):1074-82. doi: 10.1111/tri.13886 [published Online First: 2021/04/23]

9. Bezerra R, Teles F, Mendonca PB, et al. Outcomes of critically ill patients with acute kidney injury in COVID-19 infection: an observational study. *Renal failure* 2021;43(1):911-18. doi: 10.1080/0886022x.2021.1933530 [published Online First: 2021/06/01]

10. Bhandari G, Tiwari V, Gupta A, et al. COVID-19 Infection in Renal Transplant Patients: Early Report From India. *Indian journal of nephrology* 2021;31(3):271-75. doi: 10.4103/ijn.IJN_323_20 [published Online First: 2021/08/12]

11. Blom KB, Åsberg A, Sjaastad I, et al. Kidney Transplant Recipient Behavior During the Early COVID-19 Pandemic: A National Survey Study in Norway. *Kidney medicine* 2021 doi: 10.1016/j.xkme.2021.09.006 [published Online First: 2021/11/23]

12. Bonnet G, Weizman O, Trimaille A, et al. Characteristics and outcomes of patients hospitalized for COVID-19 in France: The Critical COVID-19 France (CCF) study. *Archives of cardiovascular diseases* 2021;114(5):352-63. doi: 10.1016/j.acvd.2021.01.003 [published Online First: 2021/06/23]

13. Bowe B, Cai M, Xie Y, et al. Acute Kidney Injury in a National Cohort of Hospitalized US Veterans with COVID-19. *Clinical journal of the American Society of Nephrology : CJASN* 2020;16(1):14-25. doi: 10.2215/cjn.09610620 [published Online First: 2020/11/18]

14. Caillard S, Chavarot N, Francois H, et al. Is COVID-19 infection more severe in kidney transplant recipients? *American journal of transplantation : official journal of the American Society of Transplantation and the American Society of Transplant Surgeons* 2021;21(3):1295-303. doi: 10.1111/ajt.16424 [published Online First: 2020/12/02]

15. Canevelli M, Palmieri L, Raparelli V, et al. COVID-19 mortality among migrants living in Italy. *Annali dell'Istituto superiore di sanita* 2020;56(3):373-77. doi: 10.4415/ann_20_03_16 [published Online First: 2020/09/23]

16. Carson RC, Forzley B, Thomas S, et al. Balancing the Needs of Acute and Maintenance Dialysis Patients during the COVID-19 Pandemic: A Proposed Ethical Framework for Dialysis Allocation. *Clinical journal of the American Society of Nephrology : CJASN* 2021;16(7):1122-30. doi: 10.2215/cjn.07460520 [published Online First: 2021/02/10]

17. Casas-Aparicio GA, León-Rodríguez I, Alvarado-de la Barrera C, et al. Acute kidney injury in patients with severe COVID-19 in Mexico. *PloS one* 2021;16(2):e0246595. doi: 10.1371/journal.pone.0246595 [published Online First: 2021/02/09]

18. Chan L, Chaudhary K, Saha A, et al. AKI in Hospitalized Patients with COVID-19. *Journal of the American Society of Nephrology : JASN* 2021;32(1):151-60. doi: 10.1681/asn.2020050615 [published Online First: 2020/09/05]

19. Charoenngam N, Ilori TO, Holick MF, et al. Self-identified Race and COVID-19-Associated Acute Kidney Injury and Inflammation: a Retrospective Cohort Study of Hospitalized Inner-City COVID-19 Patients. *Journal of general internal medicine* 2021;36(11):3487-96. doi: 10.1007/s11606-021-06931-1 [published Online First: 2021/06/09]

20. Chavarot N, Gueguen J, Bonnet G, et al. COVID-19 severity in kidney transplant recipients is similar to nontransplant patients with similar comorbidities. *American journal of transplantation : official journal of the American Society of Transplantation and the American Society of Transplant Surgeons* 2021;21(3):1285-94. doi: 10.1111/ajt.16416 [published Online First: 2020/12/01]

21. Chen J, Bai H, Liu J, et al. Distinct Clinical Characteristics and Risk Factors for Mortality in Female Inpatients With Coronavirus Disease 2019 (COVID-19): A Sex-stratified, Large-scale Cohort Study in Wuhan, China. *Clinical infectious diseases : an official publication of the Infectious Diseases Society of America* 2020;71(12):3188-95. doi: 10.1093/cid/ciaa920 [published Online First: 2020/07/08]

22. Chen K, Lei Y, He Y, et al. Clinical outcomes of hospitalized COVID-19 patients with renal injury: a multi-hospital observational study from Wuhan. *Scientific reports* 2021;11(1):15205. doi: 10.1038/s41598-021-94570-1 [published Online First: 2021/07/28]

23. Chilimuri S, Sun H, Alemam A, et al. Predictors of Mortality in Adults Admitted with COVID-19: Retrospective Cohort Study from New York City. *The western journal of emergency medicine* 2020;21(4):779-84. doi: 10.5811/westjem.2020.6.47919 [published Online First: 2020/07/30]

24. Chudasama YV, Zaccardi F, Gillies CL, et al. Patterns of multimorbidity and risk of severe SARS-CoV-2 infection: an observational study in the U.K. *BMC infectious diseases* 2021;21(1):908. doi: 10.1186/s12879-021-06600-y [published Online First: 2021/09/06]

25. Cohen DE, Sibbel S, Marlowe G, et al. Antibody Status, Disease History, and Incidence of SARS-CoV-2 Infection Among Patients on Chronic Dialysis. *Journal of the American Society of Nephrology : JASN* 2021;32(8):1880-86. doi: 10.1681/asn.2021030387 [published Online First: 2021/07/04]

26. Corbett RW, Blakey S, Nitsch D, et al. Epidemiology of COVID-19 in an Urban Dialysis Center. *Journal of the American Society of Nephrology : JASN* 2020;31(8):1815-23. doi: 10.1681/asn.2020040534 [published Online First: 2020/06/21]

27. Costa RLD, Sória TC, Salles EF, et al. Acute kidney injury in patients with Covid-19 in a Brazilian ICU: incidence, predictors and in-hospital mortality. *Jornal brasileiro de nefrologia : 'orgao oficial de Sociedades Brasileira e Latino-Americana de Nefrologia* 2021;43(3):349-58. doi: 10.1590/2175-8239-jbn-2020-0144 [published Online First: 2021/02/12]

28. Cristelli MP, Sandes-Freitas TV, Viana LA, et al. Migratory pattern of the coronavirus disease 2019 and high fatality rates among kidney transplant recipients: report from the Brazilian Multicenter Cohort Study. *Jornal brasileiro de nefrologia : 'orgao oficial de Sociedades Brasileira e Latino-Americana de Nefrologia* 2021 doi: 10.1590/2175-8239-jbn-2021-0063 [published Online First: 2021/07/31]

29. Dai Y, Liu Z, Du X, et al. Acute Kidney Injury in Hospitalized Patients Infected with COVID-19 from Wuhan, China: A Retrospective Study. *BioMed research international* 2021;2021:6655185. doi: 10.1155/2021/6655185 [published Online First: 2021/01/29]

30. de Almeida DC, Franco M, Dos Santos DRP, et al. Acute kidney injury: Incidence, risk factors, and outcomes in severe COVID-19 patients. *PloS one* 2021;16(5):e0251048. doi: 10.1371/journal.pone.0251048 [published Online First: 2021/05/26]

31. de Sandes-Freitas TV, de Andrade LGM, Moura LRR, et al. Comparison of 30-day case-fatality rate between dialysis and transplant Covid-19 patients: a propensity score matched cohort study. *Journal of nephrology* 2022;35(1):131-41. doi: 10.1007/s40620-021-01172-1 [published Online First: 2021/10/23]

32. Diebold M, Schaub S, Landmann E, et al. Acute kidney injury in patients with COVID-19: a retrospective cohort study from Switzerland. *Swiss medical weekly* 2021;151:w20482. doi: 10.4414/smw.2021.20482 [published Online First: 2021/03/12]

33. Doher MP, Torres de Carvalho FR, Scherer PF, et al. Acute Kidney Injury and Renal Replacement Therapy in Critically Ill COVID-19 Patients: Risk Factors and Outcomes: A Single-Center Experience in Brazil. *Blood purification* 2021;50(4-5):520-30. doi: 10.1159/000513425 [published Online First: 2020/12/21]

34. Elec AD, Oltean M, Goldis P, et al. COVID-19 after kidney transplantation: Early outcomes and renal function following antiviral treatment. *International journal of infectious diseases : IJID : official publication of the International Society for Infectious Diseases* 2021;104:426-32. doi: 10.1016/j.ijid.2021.01.023 [published Online First: 2021/01/17]

35. Esme M, Koca M, Dikmeer A, et al. Older Adults With Coronavirus Disease 2019: A Nationwide Study in Turkey. *The journals of gerontology Series A, Biological sciences and medical sciences* 2021;76(3):e68-e75. doi: 10.1093/gerona/glaa219 [published Online First: 2020/09/02]

36. Fang L, Xie H, Liu L, et al. Early predictors and screening tool developing for severe patients with COVID-19. *BMC infectious diseases* 2021;21(1):1040. doi: 10.1186/s12879-021-06662-y [published Online First: 2021/10/09]

37. Fang Z, Gao C, Cai Y, et al. A validation study of UCSD-Mayo risk score in predicting hospital-acquired acute kidney injury in COVID-19 patients. *Renal failure* 2021;43(1):1115-23. doi: 10.1080/0886022x.2021.1948429 [published Online First: 2021/07/09]

38. Fernández P, Saad EJ, Douthat Barrionuevo A, et al. The incidence, risk factors and impact of acute kidney injury in hospitalized patients due to COVID-19. *Medicina* 2021;81(6):922-30. [published Online First: 2021/12/08]

39. Flythe JE, Assimon MM, Tugman MJ, et al. Characteristics and Outcomes of Individuals With Pre-existing Kidney Disease and COVID-19 Admitted to Intensive Care Units in the United States. *American journal of kidney diseases : the official journal of the National Kidney Foundation* 2021;77(2):190-203.e1. doi: 10.1053/j.ajkd.2020.09.003 [published Online First: 2020/09/23]

40. Fominskiy EV, Scandroglio AM, Monti G, et al. Prevalence, Characteristics, Risk Factors, and Outcomes of Invasively Ventilated COVID-19 Patients with Acute Kidney Injury and Renal Replacement Therapy. *Blood purification* 2021;50(1):102-09. doi: 10.1159/000508657 [published Online First: 2020/07/14]

41. Fried MW, Crawford JM, Mospan AR, et al. Patient Characteristics and Outcomes of 11 721 Patients With Coronavirus Disease 2019 (COVID-19) Hospitalized Across the United States. *Clinical infectious diseases : an official publication of the Infectious Diseases Society of America* 2021;72(10):e558-e65. doi: 10.1093/cid/ciaa1268 [published Online First: 2020/08/29]

42. Ge E, Li Y, Wu S, et al. Association of pre-existing comorbidities with mortality and disease severity among 167,500 individuals with COVID-19 in Canada: A population-based cohort study. *PloS one* 2021;16(10):e0258154. doi: 10.1371/journal.pone.0258154 [published Online First: 2021/10/06]

43. Giusti S, Chazin S, Vaitla P, et al. Observational Study of the Clinical Characteristics and Short-Term Outcomes of Kidney Transplant Recipients Diagnosed With COVID-19 Infection (SARS-CoV-2) Requiring Hospitalization in New Orleans. *The Ochsner journal* 2021;21(4):329-34. doi: 10.31486/toj.21.0008 [published Online First: 2022/01/06]

44. Godara S, Saraf KK, Sadasukhi TC, et al. COVID-19 Infection in Kidney Transplant Recipients: A Single Centre Study from Northern India. *Indian journal of nephrology* 2021;31(6):531-35. doi: 10.4103/ijn.IJN_571_20 [published Online First: 2022/01/25]

45. Hägg S, Jylhävä J, Wang Y, et al. Age, Frailty, and Comorbidity as Prognostic Factors for Short-Term Outcomes in Patients With Coronavirus Disease 2019 in Geriatric Care. *Journal of the American Medical Directors Association* 2020;21(11):1555-59.e2. doi: 10.1016/j.jamda.2020.08.014 [published Online First: 2020/09/27]

46. He F, Luo Q, Lei M, et al. Risk factors for severe cases of COVID-19: a retrospective cohort study. *Aging* 2020;12(15):15730-40. doi: 10.18632/aging.103803 [published Online First: 2020/08/18]

47. Hectors SJ, Riyahi S, Dev H, et al. Multivariate analysis of CT imaging, laboratory, and demographical features for prediction of acute kidney injury in COVID-19 patients: a Bi-centric analysis. *Abdominal radiology (New York)* 2021;46(4):1651-58. doi: 10.1007/s00261-020-02823-w [published Online First: 2020/10/25]

48. Hernández-Galdamez DR, González-Block M, Romo-Dueñas DK, et al. Increased Risk of Hospitalization and Death in Patients with COVID-19 and Pre-existing Noncommunicable Diseases and Modifiable Risk Factors in Mexico. *Archives of medical research* 2020;51(7):683-89. doi: 10.1016/j.arcmed.2020.07.003 [published Online First: 2020/08/05]

49. Herzog AL, von Jouanne-Diedrich HK, Wanner C, et al. COVID-19 and the kidney: A retrospective analysis of 37 critically ill patients using machine learning. *PloS one* 2021;16(5):e0251932. doi: 10.1371/journal.pone.0251932 [published Online First: 2021/05/21]

50. Hilbrands LB, Duivenvoorden R, Vart P, et al. COVID-19-related mortality in kidney transplant and dialysis patients: results of the ERACODA collaboration. *Nephrology, dialysis, transplantation : official publication of the European Dialysis and Transplant Association - European Renal Association* 2020;35(11):1973-83. doi: 10.1093/ndt/gfaa261 [published Online First: 2020/11/06]

51. Islam MZ, Riaz BK, Islam A, et al. Risk factors associated with morbidity and mortality outcomes of COVID-19 patients on the 28th day of the disease course: a retrospective cohort study in Bangladesh. *Epidemiology and infection* 2020;148:e263. doi: 10.1017/s0950268820002630 [published Online First: 2020/10/30]

52. Jasuja S, Sagar G, Bahl A, et al. COVID-19 Infection Clinical Profile, Management, Outcome, and Antibody Response in Kidney Transplant Recipients: A Single Centre Experience. *International journal of nephrology* 2021;2021:3129411. doi: 10.1155/2021/3129411 [published Online First: 2021/10/08]

53. Jering KS, McGrath MM, Mc Causland FR, et al. Excess mortality in solid organ transplant recipients hospitalized with COVID-19: A large-scale comparison of SOT recipients hospitalized with or without COVID-19. *Clinical transplantation* 2022;36(1):e14492. doi: 10.1111/ctr.14492 [published Online First: 2021/09/25]

54. Jewell PD, Bramham K, Galloway J, et al. COVID-19-related acute kidney injury; incidence, risk factors and outcomes in a large UK cohort. *BMC nephrology* 2021;22(1):359. doi: 10.1186/s12882-021-02557-x [published Online First: 2021/11/02]

55. Jham S, Shafiq T, Loverre F, et al. Acute kidney injury in patients admitted with COVID-19 in a south Birmingham trust. *Clinical medicine (London, England)* 2021;21(Suppl 2):13-14. doi: 10.7861/clinmed.21-2-s13 [published Online First: 2021/06/04]

56. Ji W, Huh K, Kang M, et al. Effect of Underlying Comorbidities on the Infection and Severity of COVID-19 in Korea: a Nationwide Case-Control Study. *Journal of Korean medical science* 2020;35(25):e237. doi: 10.3346/jkms.2020.35.e237 [published Online First: 2020/07/01]

57. Kamel MH, Mahmoud H, Zhen A, et al. End-stage kidney disease and COVID-19 in an urban safety-net hospital in Boston, Massachusetts. *PloS one* 2021;16(6):e0252679. doi: 10.1371/journal.pone.0252679 [published Online First: 2021/06/05]

58. Kang SH, Kim SW, Kim AY, et al. Association between Chronic Kidney Disease or Acute Kidney Injury and Clinical Outcomes in COVID-19 Patients. *Journal of Korean medical science* 2020;35(50):e434. doi: 10.3346/jkms.2020.35.e434 [published Online First: 2020/12/30]

59. Khan MS, Dogra R, Miriyala LKV, et al. Clinical characteristics and outcomes of patients with Corona Virus Disease 2019 (COVID-19) at Mercy Health Hospitals, Toledo, Ohio. *PloS one* 2021;16(4):e0250400. doi: 10.1371/journal.pone.0250400 [published Online First: 2021/04/23]

60. Khusid JA, Becerra AZ, Gallante B, et al. Cancer, Mortality, and Acute Kidney Injury among Hospitalized Patients with SARS-CoV-2 Infection. *Asian Pacific journal of cancer prevention : APJCP* 2021;22(2):517-22. doi: 10.31557/apjcp.2021.22.2.517 [published Online First: 2021/03/01]

61. Kim E, Kim YC, Park JY, et al. Evaluation of the Prognosis of COVID-19 Patients According to the Presence of Underlying Diseases and Drug Treatment. *International journal of environmental research and public health* 2021;18(10) doi: 10.3390/ijerph18105342 [published Online First: 2021/06/03]

62. Kocayiğit H, Özmen Süner K, Tomak Y, et al. Characteristics and outcomes of critically ill patients with covid-19 in Sakarya, Turkey: a single centre cohort study. *Turkish journal of medical sciences* 2021;51(2):440-47. doi: 10.3906/sag-2005-57 [published Online First: 2020/11/14]

63. Kolhe NV, Fluck RJ, Selby NM, et al. Acute kidney injury associated with COVID-19: A retrospective cohort study. *PLoS medicine* 2020;17(10):e1003406. doi: 10.1371/journal.pmed.1003406 [published Online First: 2020/10/31]

64. Li Q, Hu P, Kang H, et al. Clinical Characteristics and Short-Term Outcomes of Acute Kidney Injury Missed Diagnosis in Older Patients with Severe COVID-19 in Intensive Care Unit. *The journal of nutrition, health & aging* 2021;25(4):492-500. doi: 10.1007/s12603-020-1550-x [published Online First: 2021/04/01]

65. Li WX, Xu W, Huang CL, et al. Acute cardiac injury and acute kidney injury associated with severity and mortality in patients with COVID-19. *European review for medical and pharmacological sciences* 2021;25(4):2114-22. doi: 10.26355/eurrev_202102_25117 [published Online First: 2021/03/05]

66. Lowe R, Ferrari M, Nasim-Mohi M, et al. Clinical characteristics and outcome of critically ill COVID-19 patients with acute kidney injury: a single centre cohort study. *BMC nephrology* 2021;22(1):92. doi: 10.1186/s12882-021-02296-z [published Online First: 2021/03/17]

67. Luther T, Bülow-Anderberg S, Larsson A, et al. COVID-19 patients in intensive care develop predominantly oliguric acute kidney injury. *Acta anaesthesiologica Scandinavica* 2021;65(3):364-72. doi: 10.1111/aas.13746 [published Online First: 2020/11/16]

68. Mahavar S, Tyagi P, Agrawal A, et al. Clinical and epidemiological profile of Indian COVID-19 patients from Jaipur: a descriptive study. *Monaldi archives for chest disease = Archivio Monaldi per le malattie del torace* 2021;91(2) doi: 10.4081/monaldi.2021.1377 [published Online First: 2021/07/22]

69. Martínez-Rueda AJ, Álvarez RD, Méndez-Pérez RA, et al. Community- and Hospital-Acquired Acute Kidney Injury in COVID-19: Different Phenotypes and Dismal Prognosis. *Blood purification* 2021;50(6):931-41. doi: 10.1159/000513948 [published Online First: 2021/03/22]

70. Martinot M, Eyriey M, Gravier S, et al. Predictors of mortality, ICU hospitalization, and extrapulmonary complications in COVID-19 patients. *Infectious diseases now* 2021;51(6):518-25. doi: 10.1016/j.idnow.2021.07.002 [published Online First: 2021/07/10]

71. Mithal A, Jevalikar G, Sharma R, et al. High prevalence of diabetes and other comorbidities in hospitalized patients with COVID-19 in Delhi, India, and their association with outcomes. *Diabetes & metabolic syndrome* 2021;15(1):169-75. doi: 10.1016/j.dsx.2020.12.029 [published Online First: 2020/12/29]

72. Mohamed NE, Benn EKT, Astha V, et al. Association between chronic kidney disease and COVID-19-related mortality in New York. *World journal of urology* 2021;39(8):2987-93. doi: 10.1007/s00345-020-03567-4 [published Online First: 2021/01/23]

73. Moledina DG, Simonov M, Yamamoto Y, et al. The Association of COVID-19 With Acute Kidney Injury Independent of Severity of Illness: A Multicenter Cohort Study. *American journal of kidney diseases : the official journal of the National Kidney Foundation* 2021;77(4):490-99.e1. doi: 10.1053/j.ajkd.2020.12.007 [published Online First: 2021/01/11]

74. Mousavi Movahed SM, Akhavizadegan H, Dolatkhani F, et al. Different incidences of acute kidney injury (AKI) and outcomes in COVID-19 patients with and without non-azithromycin antibiotics: A retrospective study. *Journal of medical virology* 2021;93(7):4411-19. doi: 10.1002/jmv.26992 [published Online First: 2021/04/02]

75. Munblit D, Nekliudov NA, Bugaeva P, et al. Stop COVID Cohort: An Observational Study of 3480 Patients Admitted to the Sechenov University Hospital Network in Moscow City for Suspected Coronavirus Disease 2019 (COVID-19) Infection. *Clinical infectious diseases : an official publication of the Infectious Diseases Society of America* 2021;73(1):1-11. doi: 10.1093/cid/ciaa1535 [published Online First: 2020/10/10]

76. Murillo-Zamora E, Trujillo X, Huerta M, et al. Male gender and kidney illness are associated with an increased risk of severe laboratory-confirmed coronavirus disease. *BMC infectious diseases* 2020;20(1):674. doi: 10.1186/s12879-020-05408-6 [published Online First: 2020/09/18]

77. Na KR, Kim HR, Ham Y, et al. Acute Kidney Injury and Kidney Damage in COVID-19 Patients. *Journal of Korean medical science* 2020;35(28):e257. doi: 10.3346/jkms.2020.35.e257 [published Online First: 2020/07/21]

78. Nachega JB, Ishoso DK, Otokoye JO, et al. Clinical Characteristics and Outcomes of Patients Hospitalized for COVID-19 in Africa: Early Insights from the Democratic Republic of the Congo. *The American journal of tropical medicine and hygiene* 2020;103(6):2419-28. doi: 10.4269/ajtmh.20-1240 [published Online First: 2020/10/04]

79. Najafi N, Akbari R, Lotfi Z, et al. The Clinical Course and Prognostic Factors of COVID-19 in Patients with Chronic Kidney Disease, A Study in Six Centers. *Iranian journal of kidney diseases* 2021;15(4):279-87. [published Online First: 2021/07/20]

80. Naser MN, Al-Ghatam R, Darwish AH, et al. Risk factors, predictions, and progression of acute kidney injury in hospitalized COVID-19 patients: An observational retrospective cohort study. *PloS one* 2021;16(9):e0257253. doi: 10.1371/journal.pone.0257253 [published Online First: 2021/09/30]

81. Ng JH, Hirsch JS, Hazzan A, et al. Outcomes Among Patients Hospitalized With COVID-19 and Acute Kidney Injury. *American journal of kidney diseases : the official journal of the National Kidney Foundation* 2021;77(2):204-15.e1. doi: 10.1053/j.ajkd.2020.09.002 [published Online First: 2020/09/23]

82. Oto OA, Ozturk S, Turgutalp K, et al. Predicting the outcome of COVID-19 infection in kidney transplant recipients. *BMC nephrology* 2021;22(1):100. doi: 10.1186/s12882-021-02299-w [published Online First: 2021/03/21]

83. Öztürk S, Turgutalp K, Arıcı M, et al. Impact of hospital-acquired acute kidney injury on Covid-19 outcomes in patients with and without chronic kidney disease: a multicenter retrospective cohort study. *Turkish journal of medical sciences* 2021;51(3):947-61. doi: 10.3906/sag-2011-169 [published Online First: 2021/02/22]

84. Ozturk S, Turgutalp K, Arici M, et al. Mortality analysis of COVID-19 infection in chronic kidney disease, haemodialysis and renal transplant patients compared with patients without kidney disease: a nationwide analysis from Turkey. *Nephrology, dialysis, transplantation : official publication of the European Dialysis and Transplant Association - European Renal Association* 2020;35(12):2083-95. doi: 10.1093/ndt/gfaa271 [published Online First: 2020/12/05]

85. Paek JH, Kim Y, Park WY, et al. Severe acute kidney injury in COVID-19 patients is associated with in-hospital mortality. *PloS one* 2020;15(12):e0243528. doi: 10.1371/journal.pone.0243528 [published Online First: 2020/12/10]

86. Parker K, Hamilton P, Hanumapura P, et al. Chronic anticoagulation is not associated with a reduced risk of acute kidney injury in hospitalised Covid-19 patients. *BMC nephrology* 2021;22(1):224. doi: 10.1186/s12882-021-02436-5 [published Online First: 2021/06/18]

87. Pei G, Zhang Z, Peng J, et al. Renal Involvement and Early Prognosis in Patients with COVID-19 Pneumonia. *Journal of the American Society of Nephrology : JASN* 2020;31(6):1157-65. doi: 10.1681/asn.2020030276 [published Online First: 2020/04/30]

88. Peng S, Wang HY, Sun X, et al. Early versus late acute kidney injury among patients with COVID-19-a multicenter study from Wuhan, China. *Nephrology, dialysis, transplantation : official publication of the European Dialysis and Transplant Association - European Renal Association* 2020;35(12):2095-102. doi: 10.1093/ndt/gfaa288 [published Online First: 2020/12/05]

89. Piñeiro GJ, Molina-Andújar A, Hermida E, et al. Severe acute kidney injury in critically ill COVID-19 patients. *Journal of nephrology* 2021;34(2):285-93. doi: 10.1007/s40620-020-00918-7 [published Online First: 2021/01/03]

90. Portolés J, Marques M, López-Sánchez P, et al. Chronic kidney disease and acute kidney injury in the COVID-19 Spanish outbreak. *Nephrology, dialysis, transplantation : official publication of the European Dialysis and Transplant Association - European Renal Association* 2020;35(8):1353-61. doi: 10.1093/ndt/gfaa189 [published Online First: 2020/09/02]

91. Rahimzadeh H, Kazemian S, Rahbar M, et al. The Risk Factors and Clinical Outcomes Associated with Acute Kidney Injury in Patients with COVID-19: Data from a Large Cohort in Iran. *Kidney & blood pressure research* 2021;46(5):620-28. doi: 10.1159/000517581 [published Online First: 2021/07/28]

92. Ramirez-Sandoval JC, Gaytan-Arocha JE, Xolalpa-Chávez P, et al. Prolonged Intermittent Renal Replacement Therapy for Acute Kidney Injury in COVID-19 Patients with Acute Respiratory Distress Syndrome. *Blood purification* 2021;50(3):355-63. doi: 10.1159/000510996 [published Online First: 2020/10/27]

93. Rao A, Ranka S, Ayers C, et al. Association of Kidney Disease With Outcomes in COVID-19: Results From the American Heart Association COVID-19 Cardiovascular Disease Registry. *Journal of the American Heart Association* 2021;10(12):e020910. doi: 10.1161/jaha.121.020910 [published Online First: 2021/06/11]

94. Requião-Moura LR, Sandes-Freitas TV, Viana LA, et al. High mortality among kidney transplant recipients diagnosed with coronavirus disease 2019: Results from the Brazilian multicenter cohort study. *PloS one* 2021;16(7):e0254822. doi: 10.1371/journal.pone.0254822 [published Online First: 2021/07/29]

95. Sacristán PG, García EC, Pérez EBP, et al. Risk of Severe Coronavirus Disease 2019 Infection in Kidney Transplant Recipients. *Transplantation proceedings* 2022;54(1):18-21. doi: 10.1016/j.transproceed.2021.08.060 [published Online First: 2022/01/10]

96. Samaan F, Carneiro de Paula E, de Lima Souza FBG, et al. COVID-19-associated acute kidney injury patients treated with renal replacement therapy in the intensive care unit: A multicenter study in São Paulo, Brazil. *PloS one* 2022;17(1):e0261958. doi: 10.1371/journal.pone.0261958 [published Online First: 2022/01/15]

97. Sancho Ferrando E, Hanslin K, Hultström M, et al. Soluble TNF receptors predict acute kidney injury and mortality in critically ill COVID-19 patients: A prospective observational study. *Cytokine* 2022;149:155727. doi: 10.1016/j.cyto.2021.155727 [published Online First: 2021/10/11]

98. Sang L, Chen S, Zheng X, et al. The incidence, risk factors and prognosis of acute kidney injury in severe and critically ill patients with COVID-19 in mainland China: a retrospective study. *BMC pulmonary medicine* 2020;20(1):290. doi: 10.1186/s12890-020-01305-5 [published Online First: 2020/11/11]

99. Santos CAQ, Rhee Y, Hollinger EF, et al. Comparative incidence and outcomes of COVID-19 in kidney or kidney-pancreas transplant recipients versus kidney or kidney-pancreas waitlisted patients: A single-center study. *Clinical transplantation* 2021;35(8):e14362. doi: 10.1111/ctr.14362 [published Online First: 2021/05/18]

100. Scarpioni R, Valsania T, Albertazzi V, et al. Acute kidney injury, a common and severe complication in hospitalized patients during the COVID-19 pandemic. *Journal of nephrology* 2021;34(4):1019-24. doi: 10.1007/s40620-021-01087-x [published Online First: 2021/06/20]

101. Shrivastava P, Prashar R, Khoury N, et al. Acute Kidney Injury in a Predominantly African American Cohort of Kidney Transplant Recipients With COVID-19 Infection. *Transplantation* 2021;105(1):201-05. doi: 10.1097/tp.0000000000003498 [published Online First: 2020/10/24]

102. Sindhu C, Prasad P, Elumalai R, et al. Clinical profile and outcomes of COVID-19 patients with acute kidney injury: a tertiary centre experience from South India. *Clinical and experimental nephrology* 2022;26(1):36-44. doi: 10.1007/s10157-021-02123-7 [published Online First: 2021/08/18]

103. Tehrani S, Killander A, Åstrand P, et al. Risk factors for death in adult COVID-19 patients: Frailty predicts fatal outcome in older patients. *International journal of infectious diseases : IJID : official publication of the International Society for Infectious Diseases* 2021;102:415-21. doi: 10.1016/j.ijid.2020.10.071 [published Online First: 2020/11/02]

104. Thompson JV, Meghani NJ, Powell BM, et al. Patient characteristics and predictors of mortality in 470 adults admitted to a district general hospital in England with Covid-19. *Epidemiology and infection* 2020;148:e285. doi: 10.1017/s0950268820002873 [published Online First: 2020/11/25]

105. Villa L, Krüger T, Seikrit C, et al. Time on previous renal replacement therapy is associated with worse outcomes of COVID-19 in a regional cohort of kidney transplant and dialysis patients. *Medicine* 2021;100(10):e24893. doi: 10.1097/md.0000000000024893 [published Online First: 2021/03/18]

106. Villanego F, Mazuecos A, Pérez-Flores IM, et al. Predictors of severe COVID-19 in kidney transplant recipients in the different epidemic waves: Analysis of the Spanish Registry. *American journal of transplantation : official journal of the American Society of Transplantation and the American Society of Transplant Surgeons* 2021;21(7):2573-82. doi: 10.1111/ajt.16579 [published Online First: 2021/03/24]

107. Wajekar SD, Bhat SM, Birajdar NB, et al. A Prospective Study of the Course and Outcome of COVID-19 Patients with Acute Kidney Injury Admitted in an Intensive Care Unit. *The Journal of the Association of Physicians of India* 2021;69(7):11-12. [published Online First: 2021/08/26]

108. Wang F, Ran L, Qian C, et al. Epidemiology and Outcomes of Acute Kidney Injury in COVID-19 Patients with Acute Respiratory Distress Syndrome: A Multicenter Retrospective Study. *Blood purification* 2021;50(4-5):499-505. doi: 10.1159/000512371 [published Online First: 2020/12/09]

109. Wang RR, He M, Kang Y. A risk score based on procalcitonin for predicting acute kidney injury in COVID-19 patients. *Journal of clinical laboratory analysis* 2021;35(6):e23805. doi: 10.1002/jcla.23805 [published Online First: 2021/05/26]

110. Wang T, Tang C, Chen R, et al. Clinical Features of Coronavirus Disease 2019 Patients With Mechanical Ventilation: A Nationwide Study in China. *Critical care medicine* 2020;48(9):e809-e12. doi: 10.1097/ccm.0000000000004473 [published Online First: 2020/07/04]

111. Xu J, Xie J, Du B, et al. Clinical Characteristics and Outcomes of Patients With Severe COVID-19 Induced Acute Kidney Injury. *Journal of intensive care medicine* 2021;36(3):319-26. doi: 10.1177/0885066620970858 [published Online First: 2020/12/04]

112. Yan Q, Zuo P, Cheng L, et al. Acute Kidney Injury Is Associated With In-hospital Mortality in Older Patients With COVID-19. *The journals of gerontology Series A, Biological sciences and medical sciences* 2021;76(3):456-62. doi: 10.1093/gerona/glaa181 [published Online First: 2020/08/09]

113. Yildirim C, Ozger HS, Yasar E, et al. Early predictors of acute kidney injury in COVID-19 patients. *Nephrology (Carlton, Vic)* 2021;26(6):513-21. doi: 10.1111/nep.13856 [published Online First: 2021/01/28]

114. Yilmaz G, Ebru O, Ibrahim B, et al. Assessment of clinical outcomes in renal transplant recipients with COVID-19. *Journal of medical virology* 2021;93(12):6760-64. doi: 10.1002/jmv.27271 [published Online First: 2021/08/14]

115. Yin M, Nie Y, Liu H, et al. Development of a europium nanoparticles lateral flow immunoassay for NGAL detection in urine and diagnosis of acute kidney injury. *BMC nephrology* 2022;23(1):30. doi: 10.1186/s12882-021-02493-w [published Online First: 2022/01/16]

116. Yu Y, Ge H, Wang X, et al. A survey on acute kidney injury in severely and critically ill COVID-19 patients without chronic kidney disease. *Annals of palliative medicine* 2021;10(6):6198-207. doi: 10.21037/apm-20-2170 [published Online First: 2021/05/29]

117. Zahid U, Ramachandran P, Spitalewitz S, et al. Acute Kidney Injury in COVID-19 Patients: An Inner City Hospital Experience and Policy Implications. *American journal of nephrology* 2020;51(10):786-96. doi: 10.1159/000511160 [published Online First: 2020/10/05]

118. Zheng X, Yang H, Li X, et al. Prevalence of Kidney Injury and Associations with Critical Illness and Death in Patients with COVID-19. *Clinical journal of the American Society of Nephrology : CJASN* 2020;15(11):1549-56. doi: 10.2215/cjn.04780420 [published Online First: 2020/09/19]

119. Zheng X, Zhao Y, Yang L. Acute Kidney Injury in COVID-19: The Chinese Experience. *Seminars in nephrology* 2020;40(5):430-42. doi: 10.1016/j.semnephrol.2020.09.001 [published Online First: 2020/12/19]

Supplementary Table S4. The adverse events in kidney transplant recipients with COVID-19

| Study | Number of included studies | Adverse events | Prevalence (95%CI) | I^2^ (*p* value) | Reference |
| --- | --- | --- | --- | --- | --- |
| Oltean, M., 2020 | 12 | mortality | 21.2% | NA | 76 |
| Kremer, D., 2021 | 74 | AKI | 50% (44%-56%) | 57% (<0.001) | 34 |
|  |  | mortality | 23% (20%-27%) | 78% (<0.001) |  |
| Chan, K. W., 2021 | 74 | AKI | 35.99% (26.20–45.79) | NA | 19 |
|  |  | urgent RRT | 12.65% (0.72–24.58) | NA |  |
| Ho, Q. Y., 2021 | 23 | AKI | 38.9% (30.6%–48.1%) | 9.26% | 35 |
|  |  | urgent RRT | 13% (10%-16%) | 19.65% |  |
|  |  | mortality | 21% (17%-26%) | 49.58% |  |
|  |  | critical presentation | 28% (23%-34%) | 64.08% |  |
